# Extracorporeal life support for patients with acute respiratory distress syndrome: report of a Consensus Conference

**DOI:** 10.1186/2110-5820-4-15

**Published:** 2014-05-24

**Authors:** Christian Richard, Laurent Argaud, Alice Blet, Thierry Boulain, Laetitia Contentin, Agnès Dechartres, Jean-Marc Dejode, Laurence Donetti, Muriel Fartoukh, Dominique Fletcher, Khaldoun Kuteifan, Sigismond Lasocki, Jean-Michel Liet, Anne-Claire Lukaszewicz, Hervé Mal, Eric Maury, David Osman, Hervé Outin, Jean-Christophe Richard, Francis Schneider, Fabienne Tamion

**Affiliations:** 1 Hôpitaux Universitaires Paris-Sud, Hôpital de Bicêtre, Service de Réanimation Médicale, EA 4533, Université Paris-Sud, F- 94270 Le Kremlin-Bicêtre, France; 2 Hospices Civils de Lyon, Groupement Hospitalier Edouard Herriot, Service de Réanimation Médicale, 69437 Lyon, France; 3 Département d’Anesthésie-Réanimation, Centre de Traitement des Brulés, Hôpitaux Universitaires Saint Louis, Lariboisière, Fernand-Widal, Hôpital Saint Louis, 75010 Paris, France; 4 CHR Orléans, Hôpital de La Source, Service de Réanimation Polyvalente, 45067 Orléans, France; 5 CHRU Tours, Hôpital Bretonneau, Service de Réanimation Polyvalente, 37000 Tours, France; 6 Centre de Recherche Epidémiologie et Biostatistique, INSERM U1153, Equipe Méthodes en Évaluation Thérapeutique des Maladies Chroniques, Centre Cochrane Français, Hôtel-Dieu, 75004 Paris, France; 7 CHU de Nantes, Hôpital Mère-Enfant, Réanimation Pédiatrique, 40000 Nantes, France; 8 CH Le Raincy-Montfermeil, Service de Réanimation, 93370 Montfermeil, France; 9 Hôpitaux Universitaire Est Parisien, Hôpital Tenon, Unité de Réanimation Médicochirurgicale, 75020 Paris, France; 10 Département d’Anesthésie, Hôpitaux Universitaires Paris Ile-de-France Ouest, Hôpital Raymond Poincaré, 92380 Garches, France; 11 Hôpital Emile Muller, Service de Réanimation Médicale, 68070 Mulhouse, France; 12 CHU Angers, LUNAM Université, Université d’Angers, Pole d’Anesthésie-Réanimation, 49000 Angers, France; 13 Département d’Anesthésie Réanimation - Réanimation Chirurgicale et Postopératoire, Groupe Hospitalier Saint-Louis Lariboisière Fernand-Widal, Hôpital Lariboisière, 75010 Paris, France; 14 Hôpitaux Universitaires Paris Nord Val de Seine, Hôpital Bichat, Service de Pneumologie, 75018 Paris, France; 15 Hôpitaux Universitaire Est Parisien, Hôpital Saint Antoine, Service de Réanimation Médicale, 75020 Paris, France; 16 Hôpitaux Universitaires Paris-Sud, Hôpital de Bicêtre, Service de Réanimation Médicale, 94270 Le Kremlin-Bicêtre, France; 17 Centre Hospitalier Intercommunal de Poissy Saint-Germain-en-Laye, Service de Réanimation Médico-chirurgicale, 78300 Poissy, France; 18 Hospices Civils de Lyon, Hôpital de la Croix-Rousse, Service de Réanimation Médicale, 69004 Lyon, France; 19 Hôpitaux Universitaires de Strasbourg et Faculté de Médecine, Université de Strasbourg, Hôpital de Hautepierre, Service de Réanimation Médicale, Lyon, France; 20 CHU Charles Nicolle, Service de Réanimation Médicale, Inserm U1096, IRIB, Université de Rouen, 76031 Rouen, France

**Keywords:** Extracorporeal life support, Extracorporeal membrane oxygenation, Extracorporeal CO_2_ removal, Acute respiratory distress syndrome, Protective ventilation

## Abstract

The influenza H1N1 epidemics in 2009 led a substantial number of people to develop severe acute respiratory distress syndrome and refractory hypoxemia. In these patients, extracorporeal membrane oxygenation was used as rescue oxygenation therapy. Several randomized clinical trials and observational studies suggested that extracorporeal membrane oxygenation associated with protective mechanical ventilation could improve outcome, but its efficacy remains uncertain. Organized by the *Société de Réanimation de Langue Française* (SRLF) in conjunction with the *Société Française d’Anesthésie et de Réanimation* (SFAR), the *Société de Pneumologie de Langue Française* (SPLF), the *Groupe Francophone de Réanimation et d’Urgences Pédiatriques* (GFRUP), the *Société Française de Perfusion* (SOFRAPERF), the *Société Française de Chirurgie Thoracique et Cardiovasculaire* (SFCTV) et the *Sociedad Española de Medecina Intensiva Critica y Unidades Coronarias* (SEMICYUC), a Consensus Conference was held in December 2013 and a jury of 13 members wrote 65 recommendations to answer the five following questions regarding the place of extracorporeal life support for patients with acute respiratory distress syndrome: 1) What are the available techniques?; 2) Which patients could benefit from extracorporeal life support?; 3) How to perform extracorporeal life support?; 4) How and when to stop extracorporeal life support?; 5) Which organization should be recommended? To write the recommendations, evidence-based medicine (GRADE method), expert panel opinions, and shared decisions taken by all the thirteen members of the jury of the Consensus Conference were taken into account.

## Introduction

The mortality of acute respiratory distress syndrome (ARDS) remains high in its severe forms as defined at the recent Berlin Consensus Conference [1]. One explanation for this is that some patients remain severely hypoxemic despite mechanical ventilation conducted according to current international recommendations including, in particular, the use of a tidal volume of between 4 and 8 mL/kg, alveolar recruitment by use of high positive expiratory pressure, prone positioning and early administration of a neuromuscular blocking agent [2]. These patients often also present respiratory acidosis related to the severity of decreased pulmonary compliance, which sometimes necessitates further reduction in tidal volume or positive expiratory pressure or both so as to keep plateau pressure < 30 cmH_2_O.

Since the 1980s, extracorporeal life support techniques (extracorporeal membrane oxygenation, ECMO) have been proposed as a means of achieving pulmonary function recovery in severe acute respiratory failure [3]. Very poor results and a highly unfavorable risk-benefit ratio were seen in early studies, for two main reasons [3,4]: the extent of complications, particularly hemorrhagic, and the use of mechanical ventilation modalities which, at that time, took insufficient account of the risk of pulmonary baro- and volutrauma.

More recently, in the 2009 influenza A (H1N1) pandemic, the occurrence of severe rapid-onset ARDS causing refractory hypoxemia prompted resurgence in the use of ECMO in combination with protective ventilation [5-8]. Data from national and international registers suggested that early use of ECMO gives favorable results in severe hypoxemia [5-8].

The availability of extracorporeal life support equipment, usually in centers with a cardiac and/or thoracic surgery department familiar with its use in daily cardiology indications, led to the suggestion that ECMO, in particular venovenous (VV ECMO), should henceforth be included in the treatment algorithm for ARDS [2,9,10].

In view of the rarity of indications, the absence of formal proof of the impact on prognosis, coupled with known potentially serious risks, considerable care burden, an unknown cost-benefit ratio and the absolute need for theoretical and practical training of medical and nursing staff, the *Société de Réanimation de Langue Française* (SRLF) held a Consensus Conference to define the modalities for use of extracorporeal life support techniques. The different disciplines involved in the use of these techniques during ARDS were represented through the participation of the *Société Française d’Anesthésie et de Réanimation* (SFAR), the *Société de Pneumologie de Langue Française* (SPLF), the *Groupe Francophone de Réanimation et d’Urgences Pédiatriques* (GFRUP), the *Société Française de Perfusion* (SOFRAPERF), the *Société Française de Chirurgie Thoracique et Cardiovasculaire* (SFCTV) et the *Sociedad Española de Medecina Intensiva Critica y Unidades Coronarias* (SEMICYUC). Taking into account also the development of extracorporeal CO_2_ removal [11,12], the current introduction of which in France seems to be uncoordinated, and to lack evaluation, the SRLF tasked the conference expert panel with drawing up recommendations based on the following five questions:

● What extracorporeal life support techniques are available?

● Which patients require extracorporeal life support?

● How is extracorporeal life support performed?

● When and how should extracorporeal life support be stopped?

● How should it be organized?

In formulating these recommendations, account was taken of literature data (gathered by a Cochrane-like review), data from experts and participants at the public meeting and the opinion of the expert panel. In most cases this enabled a consensus on the recommendations to be reached.

## Recommendations of the jury: Extracorporeal life support for patients with acute respiratory distress syndrome

1: ** What extracorporeal life support techniques are available?**

1.1 High-flow ECMO during conventional protective ventilation improves oxygenation in cases of refractory hypoxemia and/or corrects hypercapnia

1.2 High-flow ECMO enables implementation of ultra-protective ventilation strategies

1.3 Low-flow extracorporeal CO_2_ removal (ECCO_2_R) enables implementation of ultra-protective ventilation strategies

1.4 In ECMO, the flow rate at the drainage cannula is an essential determinant of oxygenation efficiency

1.5 Insertion and use of dual-lumen cannulae raises the risk of myocardial perforation

1.6 In ECMO, the choice of insertion site and of cannula diameters should enable generation of a flow rate appropriate to the treatment goals

1.7 In VV ECMO in adults, the femoral-internal jugular configuration should be preferred to generate a flow rate appropriate to the treatment goals (CR)

1.8 In ECMO, nonocclusive centrifugal pumps should be preferred (CR)

1.9 In ECMO, a polymethylpentene membrane oxygenator should be preferred (CR)

2: **Which patients require extracorporeal life support in ARDS?**

2.1 The indications for ECMO must be based on a collective and multidisciplinary decision, noted in the medical records (CR)

2.2 In ARDS, the indications for ECMO should be discussed case by case, taking into account the risk-benefit ratio

2.3 Before implementation of ECMO in ARDS, the patient should be given information and his or her consent, or that of a proxy, should be obtained

2.4 When the patient is not in a condition to express his/her wishes, and excepting emergency situations, the patient’s family/friends should be given information before implementation of ECMO in ARDS

2.5 The predictable reversibility of lung lesions and the absence of any other therapeutic limitation are indispensable prerequisites to the use of ECMO (CR)

2.6 When ARDS is severe, ECMO should not be used unless protective ventilation, where possible using prone positioning, has been implemented

2.7 In children, when the PaO_2_ is unavailable, the severity of ARDS can be assessed using the SpO_2_/FiO_2_ ratio (CR)

2.8 Among extracorporeal life support techniques, VV ECMO is the reference in severe ARDS

2.9 Current scientific knowledge precludes use of low-flow CO_2_ removal techniques (ECCO_2_R) in ARDS

2.10 Low-flow CO_2_ removal techniques (ECCO_2_R) should be assessed in clinical trials (CR)

2.11 Use of VV ECMO should be considered if the PaO_2_/FiO_2_ ratio is below 50 mmHg when FiO_2_ = 1 for at least three hours, despite a protective ventilation strategy (involving use of prone positioning) (CR)

2.12 Use of VV ECMO should be discussed if the PaO_2_/FiO_2_ ratio is below 80 mmHg when FiO_2_ = 1 for more than six hours, despite a protective ventilation strategy (involving use of prone positioning) (CR)

2.13 Use of VV ECMO should be discussed if, associated with a protective ventilation strategy (involving use of prone positioning), there is respiratory acidosis with a pH < 7.20 for over six hours (CR)

2.14 There is no indication for VA ECMO in ARDS when respiratory failure is isolated. VA ECMO can be considered if there is concurrent cardiogenic shock (CR)

2.15 When acute cor pulmonale prompts use of ECMO, it is not a mandatory indication for VA ECMO (CR)

2.16 The impossibility of using anticoagulation treatment is a classic contraindication to ECMO (CR)

2.17 The risk-benefit ratio of ECMO in ARDS should be considered unfavorable in cases of 1) hemorrhagic or potentially hemorrhagic intracranial lesions, 2) coma following cardiac arrest, 3) ARDS in which mechanical ventilation exceeds seven days, 4) severe immunosuppression, 5) multiorgan failure syndrome (SOFA > 15) (CR)

3: ** How should extracorporeal life support be implemented in ARDS?**

3.1 The setting up, priming and daily management of ECMO should be formalized, and safety check-lists should be used (CR)

3.2 For the implementation and management of ECMO, medical and nursing personnel trained in setting up the circuit should be present (CR)

3.3 In ECMO, mechanical ventilation should be adjusted to minimize plateau pressure while administering a minimum positive expiratory pressure

3.4 In percutaneous cannulation, emergency access to skilled thoracic and vascular surgeons should be organized (CR)

3.5 Ultrasound guidance should be used during percutaneous ECMO cannulation

3.6 ECMO cannulae placement should be checked by ultrasound and chest radiography (CR)

3.7 For optimal oxygenation in VV ECMO, the pump blood flow should be ≥ 60% of the theoretical cardiac output

3.8 The oxygen fraction delivered by the extracorporeal circuit (F_EC_O_2_) should give an arterial oxygen saturation SaO_2_ ≥ 88%

3.9 The sweep gas rate should give a PaCO_2_ between 30 and 40 mmHg

3.10 Anticoagulation using unfractionated heparin should be instituted to achieve an activated partial thromboplastin time (aPTT) 1.2 to 1.5 times control or an anti-Xa activity between 0.2 and 0.4 IU/mL) (CR)

3.11 If there is significant bleeding, anticoagulation should be reduced or stopped. Continuation of ECMO should be discussed (CR)

3.12 In children, precise monitoring of hemostasis is necessary and should include thromboelastography and anti-Xa activity assay (CR)

3.13 In ECMO, when the pump blood flow drops despite maintaining pump speed, check for a reduction in preload or an increase in afterload

3.14 When implementing VV ECMO for ARDS, hypoxemia may worsen or persist because of reduced hypoxic pulmonary vasoconstriction, but should prompt a search for a mechanical cause (oxygenator failure, insufficient flow, recirculation) (CR)

3.15 When implementing VV ECMO for ARDS, hypoxemia may worsen because of worsening ARDS, but should prompt a search for a mechanical cause (oxygenator failure, insufficient flow, recirculation) (CR)

4: ** When and how should extracorporeal circulation be discontinued in ARDS?**

4.1 The question of weaning from ECMO should be posed daily (CR)

4.2 When pursuing ECMO, the question of unreasonable obstinacy should be discussed at collective and multidisciplinary meetings (CR)

4.3 The discontinuation of ECMO is strongly recommended if there is a severe hemorrhagic or embolic cerebral complication (CR)

4.4 In children, the modalities of weaning from ECMO are no different from those in adults (CR)

4.5 The procedure for weaning from ECMO comprises daily checking of criteria indicative of recovery from respiratory or cardiorespiratory failure (CR)

4.6 On weaning from ECMO, the absence of acute cor pulmonale should be confirmed (CR)

4.7 The decision to discontinue ECMO is based on the results of formalized weaning over several hours (CR)

4.8 When weaning from VA ECMO, the oxygenator gas flow should be maintained (CR)

4.9 In ARDS, if weaning from VA ECMO is not possible, the possibility of a switch to VV ECMO should be considered (CR)

4.10 Anticoagulation should be stopped at least one hour before removal of cannulae (CR)

4.11 Cannulae can be removed in the operating theater or in intensive care (CR)

4.12 Removal of an arterial cannula is always a surgical procedure. Removal of a venous cannula can be medical or surgical (CR)

4.13 In children, removal of venous or arterial cannulae is always surgical (CR)

5: ** What organization is needed?**

5.1 As for quality and safety of care, a structured national organization is indispensable for optimal management of ARDS patients requiring ECMO (CR)

5.2 Epidemic conditions apart, national organization should enable the management of a least 300 patients a year (CR)

5.3 Implementation of ECMO should be part of the institutional medical plan (CR)

5.4 Identification in each region of at least one referral center possessing all human and material means essential to the care of ARDS patients and to the setting up and use of extracorporeal life support techniques: critical care, cardiac surgery and a circulatory support mobile unit (CR)

5.5 Referral centers must have a circulatory support mobile unit available 24/7 and ready to intervene in all healthcare centers in the region concerned (CR)

5.6 Each intensive care department must enter in a national register all patients treated for severe ARDS (CR)

5.7 Each intensive care department must be organized through agreement to ensure the care of ARDS patients requiring ECMO in each region

5.8 Identification in each region of at least one intensive care department able to perform ECMO, within a referral center or which has an agreement with one of the referral centers (CR)

5.9 An intensive care department able to perform ECMO in ARDS must: 1) acquire and maintain specific skills, 2) have at least two trained physicians in its medical personnel, 3) have access to emergency vascular and thoracic surgery, 4) implement a regular training program for paramedical staff, 5) formalize the indications and ensure their traceability, 6) enter data in the severe ARDS registry, and 7) at least once a year during morbidity-mortality reviews analyze all the medical records of patients treated with ECMO (CR)

5.10 Maintenance of the ECMO skills of an intensive care department may be compromised if there are fewer than ten indications for ECMO annually

5.11 Pediatric care requires inter-regional organization around pediatric referral centers (specialized pediatric intensive care) with all human and material means essential for the care of ARDS patients and the setting up and use of extracorporeal life support techniques: intensive care, cardiac surgery and a circulatory support mobile unit (CR)

5.12 To expedite ECMO in children, the pediatric referral center (specialized pediatric intensive care) should be contacted early (CR)

Legend: aPPT, activated partial thromboplastin time; ARDS, acute respiratory distress syndrome; CR, consensus recommendations (recommendations that received the majority vote of the panel); ECCO_2_R, low-flow extracorporeal CO_2_ removal; ECMO, extracorporeal membrane oxygenation; F_EC_O_2_, oxygen fraction delivered by the extracorporeal circuit; SOFA, Sequential Organ Failure score; VA ECMO, venoarterial extracorporeal membrane oxygenation; VV ECMO, venovenous extra corporeal membrane oxygenation.

## Methodology

The GRADE system (Grading of Recommendations Assessment, Development and Evaluation) and a literature analysis were used to formulate the recommendations [13,14]. A literature search covering the period January 1994 to December 2013 was done using the databases MEDLINE, EMBASE and CENTRAL (Cochrane Central Register of Controlled Trials) according to a predefined strategy (Additional file 1).

Relevant studies were selected by two people independently. For each study deemed relevant, the level of proof was assessed as a function of the type of study (randomized controlled trial, cohort study, case-control study) and its methodological quality. The selected studies were then classified so as to group those that evaluated predefined outcomes, and an overall level of proof for each of these outcomes was determined taking into account the level of proof of the individual studies. A ‘strong’ level of proof enabled formulation of a ‘strong’ recommendation (must be done, must not be done, the panel recommends …). A ‘moderate’, ‘weak’ or ‘very weak’ level of proof led to the drawing up of an ‘optional’ recommendation (should probably be done, should probably not be done …).

However, the type of formulation was not chosen solely in terms of the level of proof, but also in the light of the expert panel’s analysis of the risk-benefit ratio. When the chosen formulation was not consensual, the president of the expert panel called a vote. Given the general theme of the conference and the lack of scientific proof for many of the subjects dealt with, the panel drew up ‘consensual recommendations’ put to the vote. Recommendations that received the majority vote of the panel are indicated in the text by CR. The panel often wished to put forward simple statements, without proposing actions. In such cases, the text was drafted using the formulation ‘formal’ or ‘optional’.

## Questions

### 1: What extracorporeal life support techniques are available?

#### Definitions

1.1 High-flow ECMO during conventional protective ventilation improves oxygenation in cases of refractory hypoxemia and/or corrects hypercapnia.

1.2 High-flow ECMO enables implementation of ultra-protective ventilation strategies.

1.3 Low-flow extracorporeal CO_2_ removal (ECCO_2_R) enables implementation of ultra-protective ventilation strategies.

In ARDS, several extracorporeal life support techniques meet the objectives of oxygenation or removal of CO_2_ from the blood, and limitation of mechanical ventilation-induced lung injury [9,11,15,16]. Maintained for a few days to weeks, these techniques can result in recovery of lung function in cases of reversible respiratory failure [6-8]. Correction of hypoxemia requires high ECMO flow rates of about 3 to 7 L/minute. Effective CO_2_ removal can be achieved with low flow rates of approximately 500 mL to 1,500 mL/minute [17].

High-flow ECMO improves oxygenation in cases of hypoxemia refractory to conventional ventilation techniques, using venovenous cannulation (VV ECMO) or venoarterial cannulation (VA ECMO) [9]. *In VV ECMO*, the venous blood is drained by a cannula inserted into a large vein (internal jugular or femoral), oxygenated, and reinjected into or in the immediate proximity of the right atrium using an internal jugular or femoral cannula. Drainage is usually from the femoral vein and the return into the internal jugular vein. VV ECMO can be done using a dual-lumen cannula requiring a single vascular approach [18]. *In VA ECMO*, venous blood is drained by a cannula inserted into a large vein (internal jugular or femoral), oxygenated, and reinjected via a femoral (end in the aorta) or axillary artery cannula [19].

Low-flow ECCO_2_R includes low-flow venovenous CO_2_ removal (VVCO_2_R) and arteriovenous CO_2_ removal (AVCO_2_R) [20]. It allows removal of CO_2_ from the blood and the use of ultra-protective ventilation strategies, which reduce tidal volume and the pressure generated in the pulmonary alveoli [12]. VVCO_2_R requires venous drainage (internal jugular or femoral) and return to the venous system (internal jugular or femoral) after extra-pulmonary gas exchange. This technique is like continuous hemofiltration, notably in terms of the use of catheters and not cannulae [21]. It requires a pump and an exchanger, separate or incorporated in a single unit. Flow rates are generally low (300 to 1,500 mL/minute). The most recent technologies remove CO_2_ more effectively with smaller membrane areas [11,15]. AVCO_2_R uses the blood flow of the patient, who must be able to generate an arterial-venous pressure gradient ≥ 60 mmHg. Low cardiac output compromises its use [20].

#### Technical aspects

1.4 In ECMO, the flow rate at the drainage cannula is an essential determinant of oxygenation efficiency.

1.5 Insertion and use of dual-lumen cannulae raises the risk of myocardial perforation.

1.6 In ECMO, the choice of insertion site and of cannula diameters should enable generation of a flow rate appropriate to the treatment goals.

1.7 In VV ECMO in adults, the femoral-internal jugular configuration should be preferred to generate a flow rate appropriate to the treatment goals (CR).

1.8 In ECMO, nonocclusive centrifugal pumps should be preferred (CR).

1.9 In ECMO, a polymethylpentene membrane oxygenator should be preferred (CR).

**ECMO circuit:** connected in series, a drainage cannula, a centrifugal pump, an oxygenator to oxygenate the blood and remove CO_2_, and a return line. There is also a gas blender and a heat exchanger to warm the blood. Any break in the circuit upstream of the pump will lead to air entry. In contrast, any circuit break after the pump will cause blood loss. It is inadvisable to add an access to the circuit upstream of the pump (risk of air entry) [18].

**Cannulae:** most cannulae are made of polyvinyl chloride or silicone. They are pre-heparinized or are coated to increase biocompatibility [22]. The introducer must fit the cannulae snugly to facilitate their penetration of the blood vessel. Cannulae should be positioned along the axis of the limb to avoid kinking, and fixed by at least three points. The quality of fixation should be checked at each nursing team handover. *In VV ECMO*, the flow rate at the drainage cannula is an essential determinant of oxygenation efficiency [22]. The maximum flow rate expected with a cannula is proportional to its internal radius to the power of four, and inversely proportional to its length. The flow generated must be at least 60% of the theoretical cardiac output of the patient in order to achieve sufficient saturation of arterial hemoglobin [23]. To achieve this, drainage cannulae should be 24- to 31-gauge. Cannulae should be stiffened using a metal sheath. The use of multiperforated drainage cannulae limits injury caused by endothelial suction. Flow rate is less dependent on the diameter of the return line: 16- to 23-gauge is generally sufficient to prevent the risk of hemolysis. Dual-lumen cannulae enable both drainage and return. This type of cannula, inserted into the internal jugular, drains the superior and inferior venae cavae and enables direct return to the tricuspid valve [18]. This type of cannula carries an increased risk of myocardial perforation [24]. It is essential to ensure that the guide and cannula are not in the subhepatic veins or at the level of the tricuspid valve, but in the lower vena cava. *In practice*: femoral-jugular, femoral-femoral, jugular-femoral and jugular (using dual-lumen cannulae) configurations are possible. The femoral-jugular conformation offers the best compromise between flow rate and oxygenation [22]. The tip of the drainage cannula in the femoral vein should be 5 to 10 cm from the inferior vena cava-right atrium junction. The tip of the return line, inserted into the right internal jugular, should be at the superior vena cava-right atrium junction. This configuration generally allows a pump flow rate of 5 to 6 L/minute. In the case of femoral-femoral insertion, the tip of the drainage cannula is 5 to 10 cm from the inferior vena cava-right atrium junction. The tip of the return line in the femoral artery is located in the lower part of the right atrium [22]. The femoral-femoral configuration is accompanied by substantial recirculation, which limits its value. *In VA ECMO*, the major problem of arterial cannulation is the risk of downstream ischemia due to obstruction of the common femoral artery, which necessitates placement downstream of a reperfusion device [22]. The tip of the return line in the femoral artery is positioned in the middle part of the ascending aorta. The tip of the drainage cannula should be placed in the right atrium. *In practice*: the femoral-femoral position is the simplest and most used in emergencies. Return can also be via the axillary artery [19]. This peripheral femoral-femoral access is less complex than central access, which requires a sternotomy. Peripheral VA ECMO, however, does not allow left ventricular unloading. Venous drainage being incomplete, circulation from the right atrium to the left ventricle persists and generates flow which competes with the blood reinjected in a retrograde fashion by the arterial return line. This results in perfusion of the upper part of the body (vascularized by the arteries originating in the aortic arch) by imperfectly oxygenated blood, whereas the lower part of the body receives correctly oxygenated blood from the extracorporeal circuit (Harlequin syndrome). To avoid Harlequin syndrome, left ventricular output must be reduced by inserting an additional drainage cannula (into the right ventricle, pulmonary artery or left ventricle) or by converting peripheral VA ECMO into central VA ECMO [22].

**Pumps:** there are non-occlusive and occlusive pumps [25,26]. Occlusive pumps are generally reserved for perioperative techniques in extracorporeal circulation. Non-occlusive pumps are centrifugal pumps comprising an electrically powered motor which spins a magnetic driver which in turn propels the blood by means of impeller vanes or a cone (vortex effect) or by an Archimedes screw. They are pre- and afterload-dependent and are currently the only ones used in Europe. These centrifugal pumps are driven by a magnetic force which can generate speeds of up to 7,000 rotations/minute. This magnetic force drains the venous blood and produces a centrifugal force, which ensures a continuous and non-pulsatile flow. Centrifugal pumps maintain extracorporeal life support for a prolonged period, with a low risk of blood stagnation and hemolysis [27]. The pump flow rate appropriate to the treatment goals is a function of the rotation speed, the inlet and outlet pressures and the size of the cannulae [28].

**Oxygenator:** there are two types of oxygenator; bubble oxygenators, which are used for perioperative extracorporeal circulation during heart surgery, and membrane oxygenators, which avoid hemolysis associated with bubbling [25,26]. Only membrane oxygenators are used for peripheral ECMO. There are two types of membrane: flat and tubular [28]. The former have flat silicone membranes or membranes assembled in layers. Oxygenators with tubular membranes comprising nonporous hollow fibers generally made of polymethylpentene avoid loss of plasma components. The oxygenator should be located downstream of the pump and permit gas exchange by artificially reproducing the function of the alveolar capillary membrane. It also ensures heat exchange and warms the blood of the extracorporeal circuit [25]. It has an inlet and an outlet and is connected to a gas mixer, which is used to adjust the oxygen fraction delivered by the extracorporeal circuit (F_EC_O_2_) and the gas flow (sweep gas rate). Oxygenators are tested for measurement of pressure as a function of flow rates. The pressure gradient generated by the passage of the blood through the oxygenator should be lower than 50 mmHg/L/minute. The pressure drop corresponds to the transmembrane pressure gradient. Treatment goals distinguish oxygenation and decarboxylation. Membrane performance is evaluated by the rated flow of the oxygenator which corresponds to the amount of desaturated (75%) venous blood that can be nearly fully saturated (95%) in a given time. The flow rate is adjusted to that of the assist system. Most oxygenators support sweep flow rates of 10 mL/minute to 100 L/minute [25]. The oxygenator should be placed below the level of the patient. The gas outlet port should be directed downwards to limit the risk of obstruction [25].

### Pediatric specifics

In VV ECMO in children weighing under 15 kg, drainage from the superior vena cava is recommended to obtain a better pump flow rate, because femoral drainage with a long, fine, resistant cannula in the right atrium results in a pressure drop that is deleterious for the functioning of the extracorporeal circulation [29,30]. In VA ECMO in children under 10 kg in weight, cervical cannulation is recommended [25]. For a body weight of 30 to 40 kg and above, the techniques are comparable to those used in adults.

When using a single-lumen cannula (10- to 14-gauge), it is introduced via the right internal jugular into the right atrium. Drainage and return by the same cannula is permitted by means of a non-occlusive pump and successive clamping on the drainage and injection lines [31]. The flow rates used are a little higher (about 30% of the cardiac output). Recirculation is low.

### 2: Which patients require extracorporeal life support in ARDS?

#### General principles

2.1 The indications for ECMO must be based on a collective and multidisciplinary decision, noted in the medical records (CR).

2.2 In ARDS, the indications for ECMO should be discussed case by case, taking into account the risk-benefit ratio.

2.3 Before implementation of ECMO in ARDS, the patient should be given information and his or her consent, or that of a proxy, should be obtained.

2.4 When the patient is not in a condition to express their wishes, and excepting emergency situations, the patient’s family/friends should be given information before implementation of ECMO in ARDS.

2.5 The predictable reversibility of lung lesions and the absence of any other therapeutic limitation are indispensable prerequisites to the use of ECMO (CR).

2.6 When ARDS is severe, ECMO should not be used unless protective ventilation, where possible using prone positioning, has been implemented.

2.7 In children, when the PaO_2_ is unavailable, the severity of ARDS can be assessed using the SpO_2_/FiO_2_ ratio (CR).

ARDS is a progressive inflammatory lung disease with a heterogeneous distribution of alveolar capillary membrane involvement. These lesions progressively cause gas exchange abnormalities which result in respiratory distress [10]. In addition to treatment of the cause, respiratory assistance is necessary in severe forms. However, it has been shown that mechanical ventilation itself worsens lung lesions. Experimental studies and clinical trials have shown that such ventilator-induced lung injuries can be reduced by optimizing the ventilator settings [2]. In a severe form of ARDS, it is therefore not reasonable to use extracorporeal life support unless a protective ventilation strategy has been implemented, based on [1]:

● tidal volume between 4 and 8 mL/kg of the body weight predicted by height;

● high positive end-expiratory pressure (PEEP);

● plateau pressure ≤ 30 cmH_2_O;

● neuromuscular blocking agent during the first 48 hours;

● trial of prone positioning [32].

In children weighing less than 20 kg, optimization of mechanical ventilation may require the use of the high-frequency oscillatory ventilation [33].

Given the current level of proof of clinical studies of ECMO in ARDS, of the medical and economic impact of the spread of these techniques, and of the considerable accompanying care load, the indications should be discussed case by case in the light of the individual risk-benefit ratio. Before implementing these techniques, the patient should be given information and his or her consent or that of a proxy obtained. In the case of children, the legislation (article R.1112-35 of the Public Health Code) on surgical procedures requires the authorization of the person who holds parental authority. The expected reversibility of lung lesions and the absence of any other therapeutic limitation are a *sine qua non*.

### Extracorporeal life support techniques recommended in ARDS

2.1 Among extracorporeal life support techniques, VV ECMO is the reference in severe ARDS.

2.2 Current scientific knowledge precludes use of low-flow CO_2_ removal techniques (ECCO_2_R) in ARDS.

3.3 ECCO_2_R techniques should be assessed in clinical trials (CR).

The negative results of two randomized, prospective, multicenter studies published in 1979 and 1994 [4,34] on its use in management of severe ARDS (PaO_2_/FiO_2_ < 50 mmHg) led to the abandonment of ECMO. The reasons for the failure of ECMO reported in these studies were various: late use of ECMO after a period of prolonged mechanical ventilation associated with approximately 90% mortality, use of VA ECMO with the associated risk of cerebral hypoxemia secondary to Harlequin syndrome, a high frequency of cerebral hemorrhagic complications and use of nonprotective ventilation modalities underlying a high incidence of pulmonary baro- and volutrauma [4,34].

Taking into account the concepts of lung rest and of the need to minimize intraalveolar pressure in positive pressure mechanical ventilation, numerous observational studies have reported, in particular on the initiative of Gattinoni *et al*. [3], about 50% survival [9].

More recently, the 2009 H1N1 influenza epidemic, given the very severe forms of ARDS observed in young subjects, led to improvements in equipment (cannulae, pumps and membranes), sharpened understanding of protective ventilation modalities, and renewed interest in the use of ECMO. The analysis of observational studies [5-8] suggests a beneficial effect in terms of survival, in particular for patients with severe ARDS, even though other observational studies by teams not using ECMO have reported comparable effects [35].

The only randomized, prospective study of ECMO together with protective ventilation was done by Peek *et al*. in 180 patients with severe ARDS (Murray score > 3 or pH < 7.20). It showed a significant reduction in a composite criterion combining mortality and severe handicap at six months (37 versus 53%) in patients on ECMO, compared with conventional ventilatory support [7]. Even if the protocol of this study, which included transfer to a referral center of patients in the ECMO arm and routine use of protective ventilation only in the same arm, does not allow comparison of ECMO with optimized management of ARDS, it highlights the value of resorting to a center skilled in the use not only of ECMO, but also in the treatment of severe ARDS.

This publication also underscored that a mobile team is valuable for the transfer of unstable and severely hypoxic patients and raised the question of how the volume effect impacts trained teams familiar with the data on ECMO use [7]. Despite the insufficient level of proof provided by this literature review, the jury of the Consensus Conference considers that there are consensual indications for the use of VV ECMO.

In contrast, given the clinical data currently available, ECCO_2_R is not recommended in ARDS. However, ECCO_2_R could facilitate implementation of ventilation strategies more protective than those recommended today, so as to limit lung injury further in mechanical ventilation. For example, by the use of tidal volumes < 4 mL/kg of predicted body weight and plateau pressures < 25 cmH_2_O. These indications should be assessed in prospective clinical trials [12].

### Indications for ECMO

2.1 Use of VV ECMO should be considered if the PaO_2_/FiO_2_ ratio is below 50 mmHg when FiO_2_ = 1 for at least three hours, despite a protective ventilation strategy (involving use of prone positioning) (CR).

2.2 Use of VV ECMO should be discussed if the PaO_2_/FiO_2_ ratio is below 80 mmHg when FiO_2_ = 1 for more than six hours, despite a protective ventilation strategy (involving use of prone positioning) (CR).

2.3 Use of VV ECMO should be discussed if, associated with a protective ventilation strategy (involving use of prone positioning), there is respiratory acidosis with a pH < 7.20 for over six hours (CR).

2.4 There is no indication for VA ECMO in ARDS when respiratory failure is isolated. VA ECMO can be considered if there is concurrent cardiogenic shock (CR).

2.5 When acute cor pulmonale prompts use of ECMO, it is not a mandatory indication for VA ECMO (CR).

Despite the insufficient level of proof provided by this literature review, the jury of the Consensus Conference considers that the use of VV ECMO should nonetheless be considered if hypoxemia results in a PaO_2_/FiO_2_ ratio < 50 mmHg (measured when FiO_2_ = 1) for over three hours. Given the predictable progression of lung injury and the time required to implement VV ECMO, the jury considers it reasonable to think ahead and start collective consideration of its possible use when hypoxemia persists for over six hours, with a PaO_2_/FiO_2_ ratio < 80 mmHg and/or respiratory acidosis with a pH < 7.20. The indications for VA ECMO in ARDS are extremely limited, but can be discussed when there is concurrent cardiogenic shock [36].

### Contraindications

2.1 The impossibility of using anticoagulation treatment is a classic contraindication to ECMO (CR).

2.2 The risk-benefit ratio of ECMO in ARDS should be considered unfavorable in cases of 1) hemorrhagic or potentially hemorrhagic intracranial lesions, 2) coma following cardiac arrest, 3) ARDS in which mechanical ventilation exceeds seven days, 4) severe immunosuppression, 5) multiorgan failure syndrome (Sequential Organ Failure Assessment score (SOFA) > 15) (CR).

The only absolute contraindication to ECMO is the impossibility of using anticoagulation treatment [9]. Even though the literature provides no definitive argument in favor of relative contraindications, it appears that ECMO is likely to be less beneficial in certain subgroups of patients. In collective discussion of the indication for extracorporeal life support, account should therefore be taken of the occurrence of ARDS in the following situations [28]: cerebral hemorrhage, post-cardiac arrest coma when the neurologic state can be assessed objectively, mechanical ventilation lasting > seven days, severe immunosuppression, and multiorgan failure with a SOFA score > 15.

### 3: How should extracorporeal life support be implemented in ARDS?

#### General principles

3.1 The setting up, priming and daily management of ECMO should be formalized, and safety check-lists should be used (CR).

3.2 For the implementation and management of ECMO, medical and nursing personnel trained in setting up the circuit should be present (CR).

3.3 In ECMO, mechanical ventilation should be adjusted to minimize plateau pressure while administering a minimum positive expiratory pressure.

ECMO is an exceptional procedure which requires formalized monitoring including safety check-lists use of which has proven effective in the operating theater for extracorporeal circulation procedures [37]. The main aspects of monitoring include regular checking of pump speeds and the resulting flow rates. As the pump battery allows only one hour of operation, the electricity supply should be checked regularly. The pump and the circuit are fitted with alarms that signal flow reversal. Daily checks should be made on manual cranking of the pump for use in the event of a power failure, on the availability of a pump head for emergency replacement of a malfunctioning pump, and on clamps. There should also be routine checks for complications (bleeding, cannula contamination, embolism, hemolysis).

This specific monitoring requires trained personnel able to act quickly in the event of a complication and capable of restoring an ECMO circuit at any time. As an example, highly trained personnel are required for removal of bubbles from an ECMO circuit, which is a crucial step associated with the risk of gas embolism. Ideally, these personnel should include a perfusionist used to managing ECMO circuits who will participate in the training of the medical and nursing staff.

Whatever the mode of ventilation chosen, it should be protective in ECMO. Recent data show that patients on ECMO survive better when the median plateau pressure is around 25 cm H_2_O [8]. The respiratory frequency can be lowered to under ten cycles a minute, while FiO_2_ is progressively reduced to below 0.6 (at the same time as F_EC_O_2_) [8]. In ECMO, the use of so-called ‘ultra-protective’ ventilation with volumes below 3 mL/kg should be evaluated in terms of prognosis [12].

#### Cannulation

3.4 Vascular surgeon availability should be organized (CR).

3.5 Ultrasound guidance should be used during percutaneous ECMO cannulation.

3.6 ECMO cannula placement should be checked by ultrasound and chest radiography (CR).

Two operators are required to set up VV ECMO in conditions of surgical asepsis. In the absence of a heart surgeon during percutaneous cannulation, it is necessary to have emergency access to skilled thoracic and vascular surgeons [18]. Complications, sometimes lethal (hemorrhage, misdirection, dissection, perforation), during cannulation are described in nearly 10% of cases. As regards existing recommendations for vascular puncture in intensive care, ultrasound guidance is recommended.

Surgical femoral-femoral cannulation is the reference route for VA ECMO and requires surgical skill. The complications associated with VA ECMO are numerous, and sometimes rapidly fatal, which justifies management by medical and surgical teams used to implantation techniques, but also to the management of their complications.

Ultrasound is indispensable during the setting up and implementation of ECMO [38,39]. Guide wire insertion and placement of cannulae can be monitored using transthoracic ultrasound at the bedside, thus limiting the phenomenon of recirculation linked to poor positioning. The positioning of dual-lumen cannulae is particularly difficult, and several techniques have been put forward to improve insertion and prevent complications [24,40,41]. Ultrasound is used to assess myocardial function and detect any preload dependence.

### ECMO settings

3.1 For optimal oxygenation in VV ECMO, the pump blood flow should be ≥ 60% of the theoretical cardiac output.

3.2 The oxygen fraction delivered by the extracorporeal circuit (F_EC_O_2_) should give an arterial oxygen saturation SaO_2_ ≥ 88%.

3.3 The sweep gas rate should give a PaCO_2_ between 30 and 40 mmHg.

The settings are adjusted on the pump console. Pump speed should be above 1,500 rotations/minute. Centrifugal pumps can generate speeds up to 7,000 rotations/minute. The ideal speed is between 3,000 and 3,500 rotations/minute. The pump flow rate measured by an ultrasound probe should be above 2 L/minute. At the oxygenator, the sweep gas rate, which conditions the PaCO_2_ of the patient, should be one to two times the pump flow rate [25].

### Extracorporeal circuit anticoagulation

3.1 Anticoagulation using unfractionated heparin should be instituted to achieve an activated partial thromboplastin time (aPTT) 1.2 to 1.5 times control or an anti-Xa activity between 0.2 and 0.4 IU/mL) (CR).

3.2 If there is significant bleeding, anticoagulation should be reduced or stopped. Continuation of ECMO should be discussed (CR).

3.3 In children, precise monitoring of hemostasis is necessary and should include thromboelastography and anti-Xa activity assay (CR).

The main complications of ECMO are hemorrhagic. Their incidence is close to 50% [42]. Intracranial bleeding was seen in 10 to 12% of cases where the level of anticoagulation was highest (target aPTT around twice the control) and in 2 to 4% of cases in the REVA registry (French H1N1 influenza research network on mechanical ventilation) with lower levels of anticoagulation [8]. Data on circuit coagulation are scarce, but its incidence is probably about 15 to 20% and seems to decrease notably with heparin-coated circuits.

The risk-benefit ratio favors the use of the lowest levels of anticoagulation plus high pump speeds. It is usual to use a heparin bolus during cannulation. Thromboelastography has been proposed for monitoring of coagulation, particularly in pediatrics.

ECMO, like any extracorporeal circulation, can favor production of anti-PF4 antibodies and hence acute heparin-induced thrombocytopenia [43]. Although the heparin doses are lower with ECMO, it is advisable to consider this diagnosis when there is unexplained thrombocytopenia. Even though its deficiency, which causes resistance to heparin, occurs at low frequency, antithrombin III should be assayed because of the serious consequences of circuit coagulation and the recognized value of antithrombin III replacement therapy in restoring the efficacy of heparin.

### Causes of extracorporeal circuit malfunction

3.1 In ECMO, when the pump blood flow drops despite maintaining pump speed, check for a reduction in preload or an increase in afterload.

3.2 When implementing VV ECMO for ARDS, hypoxemia may worsen or persist because of reduced hypoxic pulmonary vasoconstriction, but should prompt a search for a mechanical cause (oxygenator failure, insufficient flow, recirculation) (CR).

3.3 When implementing VV ECMO for ARDS, hypoxemia may worsen because of worsening ARDS, but should prompt a search for a mechanical cause (oxygenator failure, insufficient flow, recirculation) (CR).

The main causes of reduced preload are hypovolemia and drainage cannula malfunction (thrombosis, movement of cannulae). The main cause of increased afterload is return line malfunction (thrombosis, movement of cannulae). Hypovolemia is also identifiable by shaking of the circuit lines. Too high a flow rate can cause suction in the zones of venous drainage, resulting in damage to the vascular endothelium. The risk then is a drop in pump flow rate because of failure of the assist system.

The main causes of persistent hypoxemia are listed in Table 1. In these situations, remember that oxygen transport also depends on cardiac output and hemoglobin level, parameters which can be modified.

**Table 1:** Hypoxemia during extracorporeal membrane oxygenation (ECMO): causes and recommendations

**Causes**	**Recommendations**
Recirculation	Check the position of the cannulae
Low flow with regards to metabolic demand	Adapt the diameter of the cannulae and correct hypovolemia
Failure of the oxygenator	Measure post-oxygenator blood gases
Inhibition of hypoxic pulmonary vasoconstriction and worsening of the pulmonary shunt	Measure pre, post-oxygenator and patient blood gases
Worsening of the pulmonary disease	

### Pediatric specifics

In children weighing less than 10 kg (blood volume 80 mL/kg), priming with saline of a circuit more than 200 mL in volume may result in hemodilution. It is therefore necessary to fill the circuit with blood, or fresh plasma, or albumin [44]. Hemofiltration coupled with ECMO allows adjustment of fluid-electrolyte balance and caloric intake [45]. Monitoring of brain tissue oxygenation using near-infrared spectroscopy has been proposed [46]. The frequency of cerebrovascular accidents reported in children is higher than in adults. These may be ischemic (frequency 4%) or hemorrhagic (6%) [42]. VA ECMO is associated with more central nervous system injury than VV ECMO [47,48]. In this context, monitoring of anticoagulation by means of thromboelastography and anti-Xa activity assay is desirable. Lastly, prone positioning improves the prognosis of children on ECMO [49].

### 4: When and how should extracorporeal circulation be discontinued in ARDS?

#### General principles

4.1 The question of weaning from ECMO should be posed daily (CR).

4.2 When pursuing ECMO, the question of unreasonable obstinacy should be discussed at collective and multidisciplinary meetings (CR).

4.3 The discontinuation of ECMO is strongly recommended if there is a severe hemorrhagic or embolic cerebral complication (CR).

4.4 In children, the modalities of weaning from ECMO are no different from those in adults (CR).

ECMO is an intensive treatment requiring high levels of care and is associated with potentially serious complications. It should be used as briefly as possible and the question of lung function recovery, and hence discontinuation of ECMO, should be posed daily.

ECMO should be stopped when its continuation seems to involve unreasonable obstinacy. In this context no precise criterion for discontinuation of ECMO has been determined. It has been reported that several weeks may be necessary for recovery and normalization of pulmonary function [9,22,50]. Certain clinical criteria observed from the first days of ECMO were associated with a poor prognosis, such as no improvement in plateau pressure, blood lactate, or hemodynamic failure [8,23,51]. In the literature, ECMO is used for a mean period of two weeks, after which the question of withdrawal should take the form of a collective discussion taking into account, as when any active treatment is limited, expected reversibility of lung and/or cardiac failure and of other organ failures, the context and in exceptional circumstances the existence of alternative treatment options, such as lung transplantation. If it is decided to discontinue ECMO, the reasons should be explained to the patient’s family/proxies.

#### Practical aspects of weaning from ECMO

4.1 The procedure for weaning from ECMO comprises daily checking of criteria indicative of recovery from respiratory or cardiorespiratory failure (CR).

4.2 On weaning from ECMO, the absence of acute cor pulmonale should be confirmed (CR).

4.3 The decision to discontinue ECMO is based on the results of formalized weaning over several hours (CR).

4.4 When weaning from VA ECMO, the oxygenator gas flow should be maintained (CR).

4.5 In ARDS, if weaning from VA ECMO is not possible, the possibility of a switch to VV ECMO should be considered (CR).

Weaning from VV ECMO is done before weaning from mechanical ventilation. It would be worth assessing the value of extubation before ECMO weaning to enable earlier mobilization of patients on ECMO.

During weaning from ECMO, it is advisable to gauge the impact of changing the sweep gas rate on the blood carbon dioxide level, variations in which can be deleterious, particularly in the cerebral circulation. If hypercapnia is persistent, low-flow CO_2_ removal should be considered, but its usefulness has not been assessed.

There are no data justifying the use of corticosteroids in weaning from ECMO.

Recovery from respiratory or cardiorespiratory failure indicated by improvement in clinical (notably in respiratory system compliance), blood gas, and radiological parameters is essential before weaning from ECMO [7,16,28]

Literature data [22,51,52] show that discontinuation of VV ECMO can be considered when weaning involves reductions in:

● sweep gas flow through the membrane until it is zero;

● F_EC_O_2_ by about 21%.

A pump flow reduction involves the risk of circuit coagulation and is not, according to many experts, routinely necessary when envisaging weaning.

When the weaning trial is prolonged, a high-flow gas mixture should be passed over the oxygenator membrane for 30 seconds every hour. Discontinuation of VV ECMO is decided when hematosis and plateau pressure are deemed satisfactory (Table 2) [18,28,52,53]. Weaning from VA ECMO is less well codified, but criteria similar to those used for weaning from VV ECMO have been proposed (Table 2). Unlike VV ECMO, gas flow over the oxygenator should be maintained to avoid reinjecting venous blood into the peripheral arterial system. A residual flow rate of 1 L/minute is generally used. If the patient cannot be weaned from VA ECMO, a switch to VV ECMO should be discussed, VA ECMO being subject to more complications than VV ECMO. Whatever the type of ECMO, right ventricular dysfunction occurring during weaning should be evaluated. If weaning from VV ECMO fails because of the onset of right cardiac insufficiency, a switch to VA ECMO can be considered.

**Table 2:** Weaning from extracorporeal membrane oxygenation (ECMO)

	**Weaning trial**	**Criteria for ECMO withdrawal**
Venovenous ECMO	F_EC_O_2_ = 21%	Pplat < 25 to 30 cmH_2_O with TV around 6 ml/kg and PEEP < 12 cmH_2_O
	Sweep gas flow 1 L/minute or stopped	and PaO_2_ > 70 mmHg on FiO_2_ < 60% or PaO_2_/FiO_2_ > 200 mmHg
	Duration: several hours	and pH > 7.3 with PCO_2_ < 50 mmHg
		and no acute cor pulmonale
Arteriovenous ECMO	F_EC_O_2_ = 21%	Pplat < 25 to 30 cmH_2_O with TV around 6 ml/kg and PEEP < 12 cmH_2_O
	Sweep gas flow 1 L/minute	and PaO_2_ > 70 mmHg on FiO_2_ < 60% or PaO_2_/FiO_2_ > 200 mmHg
	Reduce pump blood flow by steps of 0.5 L/minute	and pH > 7.3 with PCO_2_ < 50 mmHg
	Duration: several hours	and no acute cor pulmonale
		without left ventricular failure:
		left ventricular ejection fraction > 25 to 30%
		velocity-time integral > 12 cm

F_EC_O_2_, oxygen fraction delivered by the extracorporeal circuit; Pplat, plateau pressure; PEEP, positive end-expiratory pressure; TV, tidal volume.

### Removal of cannulae

4.1 Anticoagulation should be stopped at least one hour before removal of cannulae (CR).

4.2 Cannulae can be removed in the operating theater or in intensive care (CR).

4.3 Removal of an arterial cannula is always a surgical procedure. Removal of a venous cannula can be medical or surgical (CR).

4.4 In children, removal of venous or arterial cannulae is always surgical (CR).

### 5: What organization is needed?

Organization at a national level is needed to enable the use of extracorporeal life support, in particular ECMO, in severe ARDS. This organization should have at least the four following objectives:

● offer access to ECMO to all patients who need it in optimal safety conditions;

● spread facilities to allow optimal coverage countrywide;

● be able to cope with a rapid and large increase in the number of patients to be treated in an epidemic;

● take into account the importance of the risk-benefit and cost-benefit ratios of ECMO.

Epidemiological data on the incidence, severity and prognosis of ARDS in France are insufficient for reliable estimation of the number of patients requiring ECMO. Expert opinion is that, epidemic conditions apart, every year five to ten patients with severe ARDS per million inhabitants require ECMO. A survey by the SRLF and the SFAR in 2012 in 116 intensive care departments showed that approximately 300 ARDS patients were treated by ECMO in 58 departments, which is consistent with the expert opinion. Lastly, an SRLF request to the national database of the PMSI showed that, although the data are difficult to interpret, in 2012 there were 679 stays in intensive care by patients diagnosed with ARDS who underwent VV ECMO or VA ECMO, which is a total of 5,325 days of treatment.

Advances in techniques and equipment suggest that VV ECMO can be performed safely by most intensive care departments already familiar with the management of other modes of extracorporeal circulation, such as dialysis and continuous hemofiltration, subject to regular specific training. However, because of the potential seriousness of complications associated with ECMO, and because of the skills needed for management of such treatment, experts recommend that patients requiring VV ECMO or VA ECMO be managed in centers with all the means needed to define the indications for ECMO and to implement and manage it in safe conditions. The centers best suited to this seem to be those caring both for severe ARDS patients treated with VV ECMO and the more numerous patients requiring VA ECMO for cardiac reasons. This presupposes that the centers have at least an intensive care department, a cardiac surgery department, and a circulatory support mobile unit. As an example, this concept of concentration of facilities was adopted by countries like the UK and Australia, but not by others, such as Germany and Japan. The literature evidence of the advantages of such centralized organization, in terms of patient outcome and cost-effectiveness ratio is, however, indirect and insufficient. As suggested in particular by the most recent pediatric studies, the number of patients treated by ECMO (volume effect associated with this centralized organization) is determinant for the acquisition of the required expertise [54,55].

Most reports indicate that circulatory support mobile units play a determinant role [56-58]. However, in France such units do not cover the whole country for adults or for children, and for the latter there are few centers that use ECMO and even fewer specialized circulatory support mobile units.

Lastly, it is essential to take into consideration the major difficulties that France would face in the event of viral pandemic. The capacity of the centers receiving patients requiring ECMO would soon be exceeded, as was reported during the 2009 H1N1 influenza epidemic [59]. In such a situation it is important that some centers quickly become operational. The proposed organization must cope with the rapid and large increase in the number of patients to be treated during an epidemic.

In view of these comments, the jury of the Consensus Conference made the following recommendations concerning the general principles of organization on a national scale and in intensive care departments for the care of ARDS patients likely to require ECMO.

### General organizational principles

5.1 As for quality and safety of care, a structured national organization is indispensable for optimal management of ARDS patients requiring ECMO (CR).

5.2 Epidemic conditions apart, national organization should enable the management of a least 300 patients a year (CR).

5.3 Implementation of ECMO should be part of the institutional medical plan (CR).

5.4 Identification in each region of at least one referral center possessing all human and material means essential to the care of ARDS patients and to the setting up and use of extracorporeal life support techniques: critical care, cardiac surgery and a circulatory support mobile unit (CR).

5.5 Referral centers must have a circulatory support mobile unit available 24/7 and ready to intervene in all healthcare centers in the region concerned (CR).

### Organization of intensive care services

5.6 Each intensive care department must enter in a national register all patients treated for severe ARDS (CR).

5.7 Each intensive care department must be organized through agreement to ensure the care of ARDS patients requiring ECMO in each region.

5.8 Identification in each region of at least one intensive care department able to perform ECMO within a referral center, or which has an agreement with one of the referral centers (CR).

5.9 An intensive care department able to perform ECMO in ARDS must: 1) acquire and maintain specific skills, 2) have at least two trained physicians in its medical personnel, 3) have access to emergency vascular and thoracic surgery, 4) implement a regular training program for paramedical staff, 5) formalize the indications and ensure their traceability, 6) enter data in the severe ARDS registry, and 7) at least once a year during morbidity-mortality reviews analyze all the medical records of patients treated with ECMO (CR).

5.10 Maintenance of the ECMO skills of an intensive care department may be compromised if there are fewer than ten indications for ECMO annually.

### Specifics of pediatric care

5.11 Pediatric care requires inter-regional organization around pediatric referral centers (specialized pediatric intensive care) with all human and material means essential for the care of ARDS patients and the setting up and use of extracorporeal life support techniques: intensive care, cardiac surgery and a circulatory support mobile unit (CR).

5.12 To expedite ECMO in children, the pediatric referral center (specialized pediatric intensive care) should be contacted early (CR).

### Perspectives

Concerning future developments, the jury of the Consensus Conference offers the following comments:

● Current lack of knowledge concerning the epidemiology of severe ARDS in France constitutes a major obstacle to the implementation of a reasoned strategy for use of extracorporeal life support techniques, notably ECMO. The jury wants to see the creation of a national registry of severe ARDS as a response to this failing.

● Randomized, prospective, multicenter studies and national registers should, in the years to come, yield the indications for VV ECMO in the management of severe ARDS.

● There is no recognized indication for ECCO_2_R, which should only be used in ARDS in clinical research by means of randomized, prospective, multicenter studies and cohort studies. Pending the results of these studies, heightened vigilance is needed before any distribution of new equipment, with CE marking but lacking evaluation of risk-benefit and cost-benefit ratios.

● The jury’s’ recommendations concerning the proposed organization are designed to ensure nationwide coverage of needs, which are limited at present because there are few indications for VV ECMO. These recommendations also proposed to create a national network able to cope with increased demand during an H1N1 influenza-like epidemic and with any broadening of indications stemming from ongoing trial results and the anticipated increase in availability of equipment and consumables.

## Abbreviations

aPTT: activated partial thromboplastin time; ARDS: acute respiratory distress syndrome; AVCO_2_R: arteriovenous CO_2_ removal; CR: consensus recommendations; ECCO_2_R: extracorporeal CO_2_ removal; ECMO: extracorporeal membrane oxygenation; F_EC_O_2_: oxygen fraction delivered by the extracorporeal circuit; GRADE: Grading of Recommendations Assessment, Development and Evaluation; PEEP: positive end-expiratory pressure; Pplat: plateau pressure; SOFA: Sequential Organ Failure Assessment; TV: tidal volume; VA: venoarterial; VV: venovenous; VVCO_2_R: venovenous CO_2_ removal.

## Competing interests

Co-investigator for EOLIA randomized trial (collaborator: Maquet): C Richard, H Outin and D Osman. All other authors declare that they have no conflicts of interest.

## Authors’ contributions

CR, LA, TB, JMD, MF, SL, JML, ACL, HM, EM, HO, FS and FT were members of the jury and contributed to data interpretation, formulation of the recommendations and writing of the manuscript. AB, LC and AD conducted all literature searches. AB and LC separately reviewed abstracts and articles and based on the selection criteria decided the suitability of the articles. AB and LC contributed to the analysis of data. LD, DF, KK, DO and JCR were members of the organizing committee and contributed to the conception of the conference, the methodology and the analysis of the output. All authors have read and approved the final manuscript.

## Authors’ information

President of the Jury: SRLF: Christian Richard (Le Kremlin-Bicêtre); Members of the Jury SRLF: Laurent Argaud (Lyon), Thierry Boulain (Orléans), Eric Maury (Paris), Hervé Outin (Poissy), Francis Schneider (Strasbourg), Fabienne Tamion (Rouen), SFAR: Sigismond Lasocki (Angers), Anne-Claire Lukaszewicz (Paris), SPLF: Muriel Fartoukh (Paris), Hervé Mal (Paris), GFRUP: Jean-Marc Dejode (Nantes), Jean-Michel Liet (Nantes); Scientific Advisors: SRLF: Claude Guérin (Lyon) SEMICYUC: Antonio Artigas (Sabadell); Organizing Committee SRLF: Laurence Donetti, présidente (Montfermeil), Khaldoun Kuteifan (Mulhouse), David Osman (Le Kremlin-Bicêtre), Jean-Christophe Richard (Lyon), SFAR: Dominique Fletcher (Paris); Bibliography: SRLF: Alice Blet (Paris), Laetitia Contentin (Tours), Centre Cochrane Français: Agnès Dechartres (Paris); Experts: SRLF: Laurent Brochard (Genève) Gilles Capellier (Besançon) Alain Combes (Paris) Luciano Gattinoni (Milan), Luc-Marie Jacquet (Bruxelles), François Lemaire (Paris), Bruno Mégarbane (Paris), Giles Peek (Leicester), Antoine Roch (Marseille), SFAR: Olivier Bastien (Lyon) SFCTCV, Guillaume Lebreton (Paris), Pascal Leprince (Paris), GFRUP: Sylvain Renolleau (Paris), SOFRAPERF: Laurence Omnes (Paris).

## Supplementary Material

Additional file 1 Additional material. Click here for file
