# Supplementary material for: Extracorporeal life support for patients with acute respiratory distress syndrome: report of a Consensus Conference
Source: Ann Intensive Care. 2014 May 24;4:15. doi: 10.1186/2110-5820-4-15 (PMC4046033; doi:10.1186/2110-5820-4-15)
Supplement: Additional file 1 — Additional material. [file 2110-5820-4-15-S1.doc]

**Extracorporeal life support for patients with acute respiratory distress syndrome: report of a Consensus Conference**

**Additional material**

1. **Research equations**

**Embase**

1. ECMO:ti,ab

2. ‘extracorporeal Membrane Oxygenation device’/exp

3. ‘extracorporeal oxygenation’/exp

4. ‘membrane Oxygenator’/exp

5. ‘membrane Oxygenator’:ti,ab

6. ‘membrane Oxygenators’:ti,ab

7. ‘membrane oxygenation’:ti,ab

8. ‘extracorporeal circulation’/exp

9. ‘extracorporeal circulation’:ti,ab

10. ‘extracorporeal CO2 removal’:ti,ab

11. ‘extracorporeal support’:ti,ab

12. ‘extracorporeal oxygenation’:ti,ab

13. ‘lung assist’:ti,ab

14. ‘mechanical circulatory support’:ti,ab

15. ECLS:ti,ab

16. ‘extracorporeal life support’:ti,ab

17. ‘ventricular assist device’:ti,ab

18. ‘membrane lung’:ti,ab

19. ‘Heart Assist Device’/exp

20. ‘Life Support System’:ti,ab

21. ‘Life Support Systems’:ti,ab

22. ECCO2R:ti,ab

23. ‘Acute lung injury’/exp

24. ‘lung injury’:ti,ab

25. ‘respiratory failure’:ti,ab

26. ‘cardiorespiratory failure’:ti,ab

27. ‘adult respiratory distress syndrome’/exp

28. ‘respiratory distress syndrome’:ti,ab

29. ‘shock lung’:ti,ab

30. ARDS:ti,ab

31. 1OR 2 OR 3 OR 4 OR 5 OR 6 OR 7 OR 8 OR 9 OR 10 OR 11 OR 12 OR 13 OR 14 OR 15 OR 16 OR 17 OR 18 OR 19 OR 20 OR 21 OR 22

32. 23 OR 24 OR 25 OR 26 OR 27 OR 28 OR 29 OR 30

33. 31 AND 32 = ECMO et SDRA

**Pubmed**

1. ECMO[tiab]

2. Extracorporeal Membrane Oxygenation[mh]

3. Oxygenators, Membrane[mh]

4. "membrane Oxygenator "[tiab]

5. "membrane Oxygenators "[tiab]

6. "membrane oxygenation "[tiab]

7. Extracorporeal circulation[mh]

8. "extracorporeal circulation "[tiab]

9. "extracorporeal CO2 removal "[tiab]

10. "extracorporeal support "[tiab]

11. "extracorporeal oxygenation "[tiab]

12. "lung assist "[tiab]

13. "mechanical circulatory support "[tiab]

14. ECLS[tiab]

15. "extracorporeal life support"[tiab]

16. "ventricular assist device"[tiab]

17. "membrane lung"[tiab]

18. Heart-Assist Devices[mh]

19. Life Support Systems[mh]

20. "Life Support System*"[tiab]

21. ECCO2R[tiab]

22. Acute lung injury[mh]

23. "lung injury"[tiab]

24. "respiratory failure"[tiab]

25. "cardiorespiratory failure"[tiab]

26. Respiratory distress syndrome[mh]

27. "respiratory distress syndrome"[tiab]

28. "shock lung"[tiab]

29. ARDS[tiab]

30. 1 OR 2 OR 3 OR 4 OR 5 OR 6 OR 7 OR 8 OR 9 OR 10 OR 11 OR 12 OR 13 OR 14 OR 15 OR 16 OR 17 OR 18 OR 19 OR 20 OR 21 = ECMO

31. 22 OR 23 OR 24 0R 25 OR 26 OR 27 OR 28 OR 29 = SDRA

32. 30 AND 31 = ECMO et SDRA

1. **References**
2. Abrams, D., Brodie, D., Combes, A., 2013. What is new in extracorporeal membrane oxygenation for ARDS in adults? Intensive Care Med 39, 2028–2030.
3. Adrian, C., Kenneth, C., 2011. Extracorporeal life support in patients with 2009 influenza a (H1N1) with acute respiratory distress syndrome: A Singapore tertiary centre experience. American Journal of Respiratory and Critical Care Medicine 183. A 1654
4. Allen, L., Bloomfield, R., Dickson, R., Noble, D.W., Park, G., 2012. Extra-corporeal membrane oxygenation (ECMO) and H1N1-variant influenza A: The Aberdeen experience. Anaesthesia 67, 808.
5. Ang, A.L., Teo, D., Lim, C.H., Leou, K.K., Tien, S.L., Koh, M.B.C., 2009. Blood transfusion requirements and independent predictors of increased transfusion requirements among adult patients on extracorporeal membrane oxygenation -- a single centre experience. Vox Sang 96, 34–43.
6. Apostolidou, S., Schaible, T., Reinshagen, K., Hien, S., Varnholt, V., Demirakca, S., 2011. Extracorporeal membrane oxygenation for pediatric respiratory failure: A single-center experience in mannheim. Intensive Care Medicine 37, S323 –.
7. Arlt, M., Philipp, A., Voelkel, S., Rupprecht, L., Mueller, T., Hilker, M., Graf, B.M., Schmid, C., 2010. Extracorporeal membrane oxygenation in severe trauma patients with bleeding shock. Resuscitation 81, 804–9.
8. Askegard-Giesmann, J.R., Besner, G.E., Fabia, R., Caniano, D.A., Preston, T., Kenney, B.D., 2010. Extracorporeal membrane oxygenation as a lifesaving modality in the treatment of  pediatric patients with burns and respiratory failure. J Pediatr Surg 45, 1330–5.
9. Bartlett, R.H., 1997. Extracorporeal Life Support Registry Report 1995. ASAIO J 43, 104–7.
10. Bartlett, R.H., Roloff, D.W., Custer, J.R., Younger, J.G., Hirschl, R.B., 2000. Extracorporeal life support: the University of Michigan experience. JAMA 283, 904–8.
11. Bastero, P., Goldsworthy, M., Best, D., Butt, W., Shekerdemian, L., 2011. Ecmo comes of age: 21 year of extracorporeal membrane oxygenation for respiratory failure beyond the neonatal period at the royal children’s hospital, Melbourne. Pediatric Critical Care Medicine 12, A59 – A60.
12. Beardsmore, C.S., Westaway, J., Killer, H., Firmin, R.K., Pandya, H., 2007. How does the changing profile of infants who are referred for extracorporeal membrane oxygenation affect their overall respiratory outcome? Pediatrics 120, e762–8.
13. Beca, J., Butt, W., 1994. Extracorporeal membrane oxygenation for refractory septic shock in children. Pediatrics 93, 726–9.
14. Beiderlinden, M., Eikermann, M., Boes, T., Breitfeld, C., Peters, J., 2006. Treatment of severe acute respiratory distress syndrome: role of extracorporeal gas exchange. Intensive Care Med 32, 1627–31.
15. Beiderlinden, M., Treschan, T., Gorlinger, K., Peters, J., 2007. Argatroban in extracorporeal membrane oxygenation. Artif Organs 31, 461–5.
16. Bein, T., Prasser, C., Philipp, A., Muller, T., Weber, F., Schlitt, H.J., Schmid, F.-X., Taeger, K., Birnbaum, D., 2004. [Pumpless extracorporeal lung assist using arterio-venous shunt in severe ARDS. Experience with 30 cases]. Anaesthesist 53, 813–9.
17. Bein, T., Weber, F., Philipp, A., Prasser, C., Pfeifer, M., Schmid, F.-X., Butz, B., Birnbaum, D., Taeger, K., Schlitt, H.J., 2006. A new pumpless extracorporeal interventional lung assist in critical hypoxemia/hypercapnia. Crit Care Med 34, 1372–7.
18. Bein, T., Zonies, D., Philipp, A., Zimmermann, M., Osborn, E.C., Allan, P.F., Nerlich, M., Graf, B.M., Fang, R., 2012. Transportable extracorporeal lung support for rescue of severe respiratory failure in combat casualties. J Trauma Acute Care Surg 73, 1450–6.
19. Belohlavek, J., Rohn, V., Kunstyr, J., Lips, M., Semrad, M., Horak, J., Mlejnsky, F., Tosovsky, J., Linhart, A., Lindner, J., 2010. Extracorporeal membrane oxygenation in acute cardiology. European Heart Journal, Supplement 12, F93.
20. Bembea, M., Mcelrath Schwartz, J., Shah, N., Lehmann, C., Kickler, T., Pronovost, P., Strouse, J.J., 2011. Coagulation monitoring during neonatal and pediatric extracorporeal membrane oxygenation (ECMO). Clinical and Translational Science 4, 121.
21. Bembea, M.M., Lee, R., Masten, D., Kibler, K.K., Lehmann, C.U., Brady, K.M., Easley, B., 2013. Magnitude of arterial carbon dioxide change at initiation of extracorporeal membrane oxygenation support is associated with survival. J Extra Corpor Technol 45, 26–32.
22. Bembea, M.M., Savage, W., Strouse, J.J., Schwartz, J.M., Graham, E., Thompson, C.B., Everett, A., 2011. Glial fibrillary acidic protein as a brain injury biomarker in children undergoing extracorporeal membrane oxygenation. Pediatr Crit Care Med 12, 572–9.
23. Bermudez, C.A., Rocha, R.V., Sappington, P.L., Toyoda, Y., Murray, H.N., Boujoukos, A.J., 2010. Initial experience with single cannulation for venovenous extracorporeal oxygenation in adults. Ann Thorac Surg 90, 991–5.
24. Beurtheret, S., Mastroianni, C., Pozzi, M., D’Alessandro, C., Luyt, C.-E., Combes, A., Pavie, A., Leprince, P., 2012. Extracorporeal membrane oxygenation for 2009 influenza A (H1N1) acute respiratory distress syndrome: single-centre experience with 1-year follow-up. Eur J Cardiothorac Surg 41, 691–5.
25. Biondi, S., Pasquini, A., Batacchi, S., Cianchi, G., Ciapetti, M., Di Valvasone, S., Bacci, S., Varegliano, L., Solaro, M., Bonizzoli, M., Spina, R., Bonacchi, M., Zagli, G., Nozzoli, C., Peris, A., Gensini, G.F., 2010. Extracorporeal life support service in a regional referral center: The Florence experience. Critical Care 14, S69.
26. Bonacchi, M., Harmelin, G., Peris, A., Sani, G., 2011. A novel strategy to improve systemic oxygenation in venovenous extracorporeal membrane oxygenation: The “(chi)-configuration”. Journal of Thoracic and Cardiovascular Surgery 142, 1197 – 1204.
27. Bonacchi, M., Spina, R., Torracchi, L., Harmelin, G., Sani, G., Peris, A., 2013. Extracorporeal life support in patients with severe trauma: An advanced treatment strategy for refractory clinical settings. J Thorac Cardiovasc Surg 145, 1617–26.
28. Bonastre, J., Suberviola, B., Pozo, J.C., Guerrero, J.E., Torres, A., Rodriguez, A., Martin-Loeches, I., 2012. [Extracorporeal lung support in patients with severe respiratory failure secondary to the 2010-2011 winter seasonal outbreak of influenza A (H1N1) in Spain]. Med Intensiva 36, 193–9.
29. Brenner, K., Baldwin, M.R., Agerstrand, C.L., Burkart, K.M., Bulman, W.A., Bacchetta, M.D., Brodie, D., 2011. Improvements in dynamic pulmonary compliance with a very low tidal volume ventilation strategy and extracorporeal membrane oxygenation for severe acute respiratory distress syndrome. American Journal of Respiratory and Critical Care Medicine 183. A 1648
30. Brochard, L., Richard, J.-C.M., Pham, T., Brun-Buisson, C., Mercat, A., 2010. Mechanical ventilation and ecmo in ards due to influenza infection: Data from the french reva-srlf-registry. Intensive Care Medicine 36, S237.
31. Brogan, T.V., Thiagarajan, R.R., Rycus, P.T., Bartlett, R.H., Bratton, S.L., 2009. Extracorporeal membrane oxygenation in adults with severe respiratory failure: a  multi-center database. Intensive Care Med 35, 2105–14.
32. Brogan, T.V., Zabrocki, L., Thiagarajan, R.R., Rycus, P.T., Bratton, S.L., 2012. Prolonged extracorporeal membrane oxygenation for children with respiratory failure. Pediatr Crit Care Med 13, e249–54.
33. Bryner, B.S., Smith, C., Cooley, E., Bartlett, R.H., Mychaliska, G.B., 2012. Extracorporeal life support for pancreatitis-induced acute respiratory distress syndrome. Ann Surg 256, 1073–7.
34. Burrell, A., Pellegrino, V., Pilcher, D., Bernard, S., 2011. Retrieval of patients with severe respiratory failure on veno-venous extra-corporeal membrane oxygenation:a critical care physician-led model. Critical Care Medicine 39, 211.
35. Burrell, A., Pellegrino, V., Pilcher, D., Bernard, S., Kennedy, M., 2012. Retrieval of patients with severe respiratory failure on venovenous extracorporeal membrane oxygenation: An intensivist-led model. Critical Care 16, S34.
36. Cambier, J.F., Bulpa, P., Bouhon, S., Gonzalez, M., Michaux, I., Installe, E., Dive, A., Evrard, P., 2009. Is mobile extracorporeal membrane oxygenation a safe tool to transfer severely hypoxaemic patients because of acute respiratory distress syndrome or cardiac failure? Intensive Care Medicine 35, S189.
37. Camboni, D., Philipp, A., Foltan, M., Ruprecht, L., Muller, T., Lubnow, M., Arlt, M., Hilker, M., Schmid, C., 2011. Institutional experience with veno-venous extracorporeal membrane oxygenation in 89 adult patients suffering from acute lung failure. Thoracic and Cardiovascular Surgeon 58. V147
38. Camboni, D., Philipp, A., Lubnow, M., Bein, T., Haneya, A., Diez, C., Schmid, C., Muller, T., 2011. Support time-dependent outcome analysis for veno-venous extracorporeal membrane oxygenation. Eur J Cardiothorac Surg 40, 1341–6;discussion 1346–7.
39. Carlese, A., Jakobleff, W., Weinstein, S., Shiloh, A., Leung, S., 2012. Using bedside transthoracic echocardiography for the placement of a double lumen bicaval venovenous extracorporeal membrane oxygenation (VV ECMO) cannula. Critical Care Medicine 40, 269.
40. Carton, E., 2011. Extracorporeal life support for severe respiratory failure. Irish Journal of Medical Science 180, S463.
41. Castillo, L., Bugedo, G., Hernandez, G., Montes, J.M., Pablo Ilic, J., Labarca, E., 1996. [Extracorporeal respiratory assistance. Experience in the treatment of severe acute respiratory failure]. Rev Med Chil 124, 45–56.
42. Chan, K.K.C., Lee, K.L., Lam, P.K.N., Law, K.I., Joynt, G.M., Yan, W.W., 2010. Hong Kong’s experience on the use of extracorporeal membrane oxygenation for the  treatment of influenza A (H1N1). Hong Kong Med J 16, 447–54.
43. Chang, W.-W., Tsai, F.-C., Tsai, T.-Y., Chang, C.-H., Jenq, C.-C., Chang, M.-Y., Tian, Y.-C., Hung, C.-C., Fang, J.-T., Yang, C.-W., Chen, Y.-C., 2012. Predictors of mortality in patients successfully weaned from extracorporeal membrane oxygenation. PLoS One 7, e42687.
44. Chen, Y.-C., Tsai, F.-C., Chang, C.-H., Lin, C.-Y., Jenq, C.-C., Juan, K.-C., Hsu, H.-H., Chang, M.-Y., Tian, Y.-C., Hung, C.-C., Fang, J.-T., Yang, C.-W., 2011. Prognosis of patients on extracorporeal membrane oxygenation: the impact of acute kidney injury on mortality. Ann Thorac Surg 91, 137–42.
45. Chiou, Y.-Y., Lee, C.-S., Hong, W.-P., Wang, J.-N., Wu, J.-M., 2009. Continuous renal replacement therapy as the supplemental therapy of extracorporeal membrane oxygenation in pediatric critical children. Hemodialysis International 13, 422.
46. Chiu, L., Chen, Y., Hu, H., Huang, C., Tsai, F., Kao, K., 2012. Resolution of organ functional scores to predict outcomes in severe acute respiratory distress syndrome patients receiving extracorporeal membrane oxygenation. Critical Care 16, S36.
47. Cho, W.H., Lee, K., Huh, J.W., Lim, C.-M., Koh, Y., Hong, S.-B., 2012. Physiologic effect and safety of the pumpless extracorporeal interventional lung  assist system in patients with acute respiratory failure--a pilot study. Artif Organs 36, 434–8.
48. Choi, N.-K., Hwang, S., Kim, K.-W., Park, G.-C., Yu, Y.-D., Jung, S.-H., Park, P.-J., Choi, Y.-I., Song, G.-W., Jung, D.-H., Hong, S.-K., Ahn, C.-S., Kim, K.-H., Moon, D.-B., Ha, T.-Y., Lee, S.-G., 2012. Intensive pulmonary support using extracorporeal membrane oxygenation in adult patients undergoing liver transplantation. Hepatogastroenterology 59, 1189–93.
49. Choptiris, S., Maggs, N., Smith, M., Vuylsteke, A., Jenkins, D., 2010. Veno-venous ecmo in management of acute respiratory failure due to H1N1/2009 influenza infection. Heart Surgery Forum 13, S91.
50. Cianchi, G., Bonizzoli, M., Pasquini, A., Bonacchi, M., Zagli, G., Ciapetti, M., Sani, G., Batacchi, S., Biondi, S., Bernardo, P., Lazzeri, C., Giovannini, V., Azzi, A., Abbate, R., Gensini, G., Peris, A., 2011. Ventilatory and ECMO treatment of H1N1-induced severe respiratory failure: results of an Italian referral ECMO center. BMC Pulm Med 11, 2.
51. Ciapetti, M., Cianchi, G., Zagli, G., Greco, C., Pasquini, A., Spina, R., Batacchi, S., Bonizzoli, M., Bonacchi, M., Lazzeri, C., Bernardo, P., Peris, A., 2011. Feasibility of inter-hospital transportation using extra-corporeal membrane oxygenation (ECMO) support of patients affected by severe swine-flu(H1N1)-related ARDS. Scand J Trauma Resusc Emerg Med 19, 32.
52. Clement, K., Fiser, R., Fiser, W., Chipman, C., Taylor, B., Heulitt, M., Moss, M., Fasules, J., Faulkner, S., Imamura, M., Fontenot, E., Jaquiss, R., 2010. Inter-hospital transport of children supported with extra-corporeal membrane oxygenation. Artificial Organs 34, A4.
53. Clement, K.C., Fiser, R.T., Fiser, W.P., Chipman, C.W., Taylor, B.J., Heulitt, M.J., Moss, M., Fasules, J.W., Faulkner, S.C., Imamura, M., Fontenot, E.E., Jaquiss, R.D.B., 2010. Single-institution experience with interhospital extracorporeal membrane oxygenation transport: A descriptive study. Pediatr Crit Care Med 11, 509–13.
54. Conrad, S., Grier, L., Scott, L., Green, R., Jordan, M., 2012. Percutaneous cannulation for extracorporeal life support by intensivists: A single institution experience. Critical Care Medicine 40, 272.
55. Conrad, S.A., Rycus, P.T., 1998. Extracorporeal life support 1997. ASAIO J 44, 848–52.
56. Conrad, S.A., Rycus, P.T., Dalton, H., 2005. Extracorporeal Life Support Registry Report 2004. ASAIO J 51, 4–10.
57. Conrad, S.A., Zwischenberger, J.B., Grier, L.R., Alpard, S.K., Bidani, A., 2001. Total extracorporeal arteriovenous carbon dioxide removal in acute respiratory failure: a phase I clinical study. Intensive Care Med 27, 1340–51.
58. Cordell-Smith, J.A., Roberts, N., Peek, G.J., Firmin, R.K., 2006. Traumatic lung injury treated by extracorporeal membrane oxygenation (ECMO). Injury 37, 29–32.
59. Cortina, G., Jungraithmayr, T., Schonlaub, J., Schermer, E., Neu, N., Fruhwirth, M., Zimmerhackl, L.B., 2010. Extracorporeal membrane oxygenation (ECMO) with or without renal replacement therapy (RRT) in a pediatric intensive care unit. Pediatric Nephrology 25, 1589.
60. Cortina, G., Schonlaub, J., Geiger, R., Schweigmann, U., Schermer, E., Neu, N., Fruhwith, M., 2011. Extracorporal membrane oxygenation (ECMO) with or without renal replacment therapy (RRT) in a pediatric intensive care unit. Pediatric Critical Care Medicine 12, A71.
61. Crossno, P.F., Miller III, R.R., Dean, N.C., Morris, A.H., Markewitz, B.A., 2010. Outcomes among pandemic 2009 novel H1N1 influenza patients fulfilling historical criteria for extracorporeal membrane oxygenation (ECMO): Salt lake county experience. American Journal of Respiratory and Critical Care Medicine 181. A 6118
62. Crucean, A.C., Peek, G.J., 2010. CESAR study: Justification of extracorporeal membrane oxygenation for severe adult respiratory failure. Etude CESAR: Justification de l’assistance respiratoire extra corporelle pour les graves syndromes de detresse respiratoire de l’adulte 31, S46 – S51.
63. D’Ancona, G., Capitanio, G., Chiaramonte, G., Serretta, R., Turrisi, M., Pilato, M., Arcadipane, A., 2011. Extracorporeal membrane oxygenator rescue and airborne transportation of patients with influenza A (H1N1) acute respiratory distress syndrome in a Mediterranean underserved area. Interact Cardiovasc Thorac Surg 12, 935–7.
64. Dagan, O., Klein, J., Bohn, D., Koren, G., 1994. Effects of extracorporeal membrane oxygenation on morphine pharmacokinetics in infants. Crit Care Med 22, 1099–101.
65. Davies, A., Jones, D., Bailey, M., Beca, J., Bellomo, R., Blackwell, N., Forrest, P., Gattas, D., Granger, E., Herkes, R., Jackson, A., McGuinness, S., Nair, P., Pellegrino, V., Pettila, V., Plunkett, B., Pye, R., Torzillo, P., Webb, S., Wilson, M., Ziegenfuss, M., 2009. Extracorporeal Membrane Oxygenation for 2009 Influenza A(H1N1) Acute Respiratory  Distress Syndrome. JAMA 302, 1888–95.
66. De Bucourt, M., Teichgraber, U.K.M., 2012. Image guided placement of extracorporeal life support through bi-caval dual lumen venovenous membrane oxygenation in an interventional radiology setting--initial experience. J Vasc Access 13, 221–5.
67. De Luca, D., Piastra, M., Chidini, G., Tissieres, P., Calderini, E., Essouri, S., Medina Villanueva, A., Vivanco Allende, A., Pons-Odena, M., Perez-Baena, L., Hermon, M., Tridente, A., Conti, G., Antonelli, M., Kneyber, M., Respiratory Section of the European Society for Pediatric Neonatal Intensive Care (ESPNIC), 2013. The use of the Berlin definition for acute respiratory distress syndrome during infancy and early childhood: multicenter evaluation and expert consensus. Intensive Care Med 39, 2083–2091.
68. Desebbe, O., Rosamel, P., Henaine, R., Vergnat, M., Farhat, F., Dubien, P.Y., Bastien, O., 2013. [Interhospital transport with extracorporeal life support: results and perspectives after 5 years experience]. Ann Fr Anesth Reanim 32, 225–30.
69. Domico, M.B., Ridout, D.A., Bronicki, R., Anas, N.G., Cleary, J.P., Cappon, J., Goldman, A.P., Brown, K.L., 2012. The impact of mechanical ventilation time before initiation of extracorporeal life support on survival in pediatric respiratory failure: a review of the Extracorporeal Life Support Registry. Pediatr Crit Care Med 13, 16–21.
70. Duke, T., Butt, W., South, M., Shann, F., 1997. The DCO2 measured by gastric tonometry predicts survival in children receiving extracorporeal life support. Comparison with other hemodynamic and biochemical information. Royal Children’s Hospital ECMO Nursing Team. Chest 111, 174–9.
71. Fallon, S.C., Shekerdemian, L.S., Olutoye, O.O., Cass, D.L., Zamora, I.J., Nguyen, T., Kim, E.S., Larimer, E.L., Lee, T.C., 2013. Initial experience with single-vessel cannulation for venovenous extracorporeal membrane oxygenation in pediatric respiratory failure*. Pediatr Crit Care Med 14, 366–73.
72. Fisher, J.C., Stolar, C.J.H., Cowles, R.A., 2008. Extracorporeal membrane oxygenation for cardiopulmonary failure in pediatric patients: is a second course justified? J Surg Res 148, 100–8.
73. Floerchinger, B., Philipp, A., Foltan, M., Rupprecht, L., Klose, A., Camboni, D., Bruenger, F., Schopka, S., Arlt, M., Hilker, M., Schmid, C., 2010. Switch from venoarterial extracorporeal membrane oxygenation to arteriovenous pumpless extracorporeal lung assist. Ann Thorac Surg 89, 125–31.
74. Florchinger, B., Philipp, A., Klose, A., Hilker, M., Kobuch, R., Rupprecht, L., Keyser, A., Puhler, T., Hirt, S., Wiebe, K., Muller, T., Langgartner, J., Lehle, K., Schmid, C., 2008. Pumpless extracorporeal lung assist: a 10-year institutional experience. Ann Thorac Surg 86, 410–7; discussion 417.
75. Foley, D.S., Pranikoff, T., Younger, J.G., Swaniker, F., Hemmila, M.R., Remenapp, R.A., Copenhaver, W., Landis, D., Hirschl, R.B., Bartlett, R.H., 2002. A review of 100 patients transported on extracorporeal life support. ASAIO J 48, 612–9.
76. Forrest, P., Ratchford, J., Burns, B., Herkes, R., Jackson, A., Plunkett, B., Torzillo, P., Nair, P., Granger, E., Wilson, M., Pye, R., 2011. Retrieval of critically ill adults using extracorporeal membrane oxygenation: an  Australian experience. Intensive Care Med 37, 824–30.
77. Fortenberry, J.D., Meier, A.H., Pettignano, R., Heard, M., Chambliss, C.R., Wulkan, M., 2003. Extracorporeal life support for posttraumatic acute respiratory distress syndrome at a children’s medical center. J Pediatr Surg 38, 1221–6.
78. Freeman, C., Bennett, T., Hubbard, A., Larsen, G., Wilkes, J., Bratton, S., 2012. Pediatric and neonatal extracorporeal membrane oxygenation; does center volume impact mortality? Critical Care Medicine 40, 10.
79. Frenckner, B., Frisen, G., Palmer, P., Linden, V., 2004. [Swedish experiences with ECMO--treatment with an artificial lung]. Lakartidningen 101, 1272–5, 1278–9.
80. Frenckner, B., Palmer, P., Linden, V., 2002. Extracorporeal respiratory support and minimally invasive ventilation in severe ARDS. Minerva Anestesiol 68, 381–6.
81. Garcia, J.P., Kon, Z.N., Evans, C., Wu, Z., Iacono, A.T., McCormick, B., Griffith, B.P., 2011. Ambulatory veno-venous extracorporeal membrane oxygenation: innovation and pitfalls. J Thorac Cardiovasc Surg 142, 755–61.
82. Gariboldi, V., Grisoli, D., Tarmiz, A., Jaussaud, N., Chalvignac, V., Kerbaul, F., Collart, F., 2010. Mobile extracorporeal membrane oxygenation unit expands cardiac assist surgical programs. Ann Thorac Surg 90, 1548–52.
83. Goettler, C.E., Pryor, J.P., Hoey, B.A., Phillips, J.K., Balas, M.C., Shapiro, M.B., 2002. Prone positioning does not affect cannula function during extracorporeal membrane oxygenation or continuous renal replacement therapy. Crit Care 6, 452–5.
84. Goldman, A.P., Kerr, S.J., Butt, W., Marsh, M.J., Murdoch, I.A., Paul, T., Firmin, R.K., Tasker, R.C., Macrae, D.J., 1997. Extracorporeal support for intractable cardiorespiratory failure due to meningococcal disease. Lancet 349, 466–9.
85. Golicnik, A., Knezevic, I., Racic, M., Noc, M., Gorjup, V., 2012. Extracorporeal life support: first year of experience implementing the technique  in Slovenia. Int J Artif Organs 35, 392–9.
86. Goretsky, M.J., Greenhalgh, D.G., Warden, G.D., Ryckman, F.C., Warner, B.W., 1995. The use of extracorporeal life support in pediatric burn patients with respiratory failure. J Pediatr Surg 30, 620–3.
87. Gow, K.W., Heiss, K.F., Wulkan, M.L., Katzenstein, H.M., Rosenberg, E.S., Heard, M.L., Rycus, P.T., Fortenberry, J.D., 2009. Extracorporeal life support for support of children with malignancy and respiratory or cardiac failure: The extracorporeal life support experience. Crit Care Med 37, 1308–16.
88. Grasso, S., Terragni, P., Birocco, A., Urbino, R., Del Sorbo, L., Filippini, C., Mascia, L., Pesenti, A., Zangrillo, A., Gattinoni, L., Ranieri, V.M., 2012. ECMO criteria for influenza A (H1N1)-associated ARDS: role of transpulmonary pressure. Intensive Care Med 38, 395–403.
89. Green, T.P., Moler, F.W., Goodman, D.M., 1995. Probability of survival after prolonged extracorporeal membrane oxygenation in pediatric patients with acute respiratory failure. Extracorporeal Life Support Organization. Crit Care Med 23, 1132–9.
90. Green, T.P., Timmons, O.D., Fackler, J.C., Moler, F.W., Thompson, A.E., Sweeney, M.F., 1996. The impact of extracorporeal membrane oxygenation on survival in pediatric patients with acute respiratory failure. Pediatric Critical Care Study Group. Crit Care Med 24, 323–9.
91. Guinot, P.-G., Zogheib, E., Detave, M., Moubarak, M., Hubert, V., Badoux, L., Bernard, E., Besserve, P., Caus, T., Dupont, H., 2011. Passive leg raising can predict fluid responsiveness in patients placed on venovenous extracorporeal membrane oxygenation. Crit Care 15, R216.
92. Guirand, D., Okoye, O., Martin, S., Aden, J., Hines, M., Pranikoff, T., Cestero, R., Inaba, K., Cannon, J., 2011. Extracorporeal life support improves survival in adult trauma patients with severe respiratory failure: A multicenter retrospective cohort study. Chest 140: 911-A
93. Gupta, M., Shanley, T.P., Moler, F.W., 2008. Extracorporeal life support for severe respiratory failure in children with immune compromised conditions. Pediatr Crit Care Med 9, 380–5.
94. Gupta, P., McDonald, R., Chipman, C.W., Stroud, M., Gossett, J.M., Imamura, M., Bhutta, A.T., 2012. 20-year experience of prolonged extracorporeal membrane oxygenation in critically ill children with cardiac or pulmonary failure. Ann Thorac Surg 93, 1584–90.
95. Haefner, S.M., Bratton, S.L., Annich, G.M., Bartlett, R.H., Custer, J.R., 2003. Complications of intermittent prone positioning in pediatric patients receiving extracorporeal membrane oxygenation for respiratory failure. Chest 123, 1589–94.
96. Han, S.J., Kim, H.S., Kim, K.I., Whang, S.M., Hong, K.S., Lee, W.K., Lee, S.H., 2011. Use of nafamostat mesilate as an anticoagulant during extracorporeal membrane oxygenation. J Korean Med Sci 26, 945–50.
97. Haneya, A., Philipp, A., Foltan, M., Camboni, D., Mueller, T., Bein, T., Schmid, C., Lubnow, M., 2012. First experience with the new portable extracorporeal membrane oxygenation system Cardiohelp for severe respiratory failure in adults. Perfusion 27, 150–5.
98. Haneya, A., Philipp, A., Foltan, M., Mueller, T., Camboni, D., Rupprecht, L., Puehler, T., Hirt, S., Hilker, M., Kobuch, R., Schmid, C., Arlt, M., 2009. Extracorporeal circulatory systems in the interhospital transfer of critically ill patients: experience of a single institution. Ann Saudi Med 29, 110–4.
99. Hanks, G.E., 1990. RE: The risk of distant metastases after transurethral resection of the prostate versus needle biopsy in patients with localized prostate cancer. J. Urol. 144, 751–752.
100. Heggen, J.A., Fortenberry, J.D., Tanner, A.J., Reid, C.A., Mizzell, D.W., Pettignano, R., 2004. Systemic hypertension associated with venovenous extracorporeal membrane oxygenation for pediatric respiratory failure. J Pediatr Surg 39, 1626–31.
101. Heilmann, C., Geisen, U., Beyersdorf, F., Nakamura, L., Benk, C., Trummer, G., Berchtold-Herz, M., Schlensak, C., Zieger, B., 2012. Acquired von Willebrand syndrome in patients with extracorporeal life support (ECLS). Intensive Care Med 38, 62–8.
102. Hemmila, M.R., Rowe, S.A., Boules, T.N., Miskulin, J., McGillicuddy, J.W., Schuerer, D.J., Haft, J.W., Swaniker, F., Arbabi, S., Hirschl, R.B., Bartlett, R.H., 2004. Extracorporeal life support for severe acute respiratory distress syndrome in adults. Ann Surg 240, 595–605; discussion 605–7.
103. Henzler, D., Dembinski, R., Kopp, R., Hawickhorst, R., Rossaint, R., Kuhlen, R., 2004. [Treatment of acute respiratory distress syndrome in a treatment center. Success  is dependent on risk factors]. Anaesthesist 53, 235–43.
104. Hermans, G., Meersseman, W., Wilmer, A., Meyns, B., Bobbaers, H., 2007. Extracorporeal membrane oxygenation: experience in an adult medical ICU. Thorac Cardiovasc Surg 55, 223–8.
105. Hermon, M., Mostafa, G., Golej, J., Burda, G., Vargha, R., Trittenwein, G., 2011. Outcome and complications in infants with respiratory failure: Venovenous two-site versus double-lumen ECMO. Critical Care 15, S60.
106. Hervey-Jumper, S.L., Annich, G.M., Yancon, A.R., Garton, H.J.L., Muraszko, K.M., Maher, C.O., 2011. Neurological complications of extracorporeal membrane oxygenation in children. J Neurosurg Pediatr 7, 338–44.
107. Hilker, M., Philipp, A., Arlt, M., Zimmermann, M., Bein, T., Schmid, C., 2011. Extracorporeal lung support in war-related lung injury. Thoracic and Cardiovascular Surgeon 59. V80
108. Hodgson, C., Carteaux, G., Tuxen, D.V., Davies, A.R., Pellegrino, V., Capellier, G., Cooper, D.J., Nichol, A., 2013. Hypoxaemic rescue therapies in acute respiratory distress syndrome: Why, when, what and which one? Injury 44, 1700–1709.
109. Hodgson, C., Hayes, K., Everard, T., Pellegrino, V., 2011. Long-term outcomes of patients receiving extracorporeal membrane oxygenation for severe acute respiratory distress syndrome. Anaesthesia and Intensive Care 39, 1145
110. Hodgson, C.L., Hayes, K., Everard, T., Nichol, A., Davies, A.R., Bailey, M.J., Tuxen, D.V., Cooper, D.J., Pellegrino, V., 2012. Long-term quality of life in patients with acute respiratory distress syndrome requiring extracorporeal membrane oxygenation for refractory hypoxaemia. Crit Care 16, R202.
111. Holzgraefe, B., Broome, M., Kalzen, H., Konrad, D., Palmer, K., Frenckner, B., 2010. Extracorporeal membrane oxygenation for pandemic H1N1 2009 respiratory failure. Minerva Anestesiol 76, 1043–51.
112. Holzgraefe, B., Broome, M., Kalzen, H., Palmer, K., Frenckner, B., 2010. Extracorporeal membrane oxygenation for severe respiratory failure related to infection with novel influenza a/H1N1. Experience from the ecmo centre Karolinska, Sweden. Intensive Care Medicine 36, S236.
113. Horne, D., Lee, J.J., Maas, M., Divekar, A., Kesselman, M., Drews, T., Veroukis, S., Hancock, B.J., Hiebert, B., Cronin, G., Soni, R., 2012. Air Transported Pediatric Rescue Extracorporeal Membrane Oxygenation: A Single Institutional Review. World Journal for Pediatric and Congenital Hearth Surgery 3, 236 – 240.
114. Hou, X., Guo, L., Zhan, Q., Jia, X., Mi, Y., Li, B., Sun, B., Hao, X., Li, H., 2012. Extracorporeal membrane oxygenation for critically ill patients with 2009 influenza A (H1N1)-related acute respiratory distress syndrome: preliminary experience from a single center. Artif Organs 36, 780–6.
115. Huang, Y.-F., Liu, P.-Y., Pan, C.-Y., Hsieh, K.-S., 2011. Poor response after pediatric extracorporeal membrane oxygenation support for severe necrotizing pneumococcus pneumonia. European Journal of Internal Medicine 22, S41.
116. Ichiba, S., Bartlett, R.H., 1996. Current status of extracorporeal membrane oxygenation for severe respiratory failure. Artif Organs 20, 120–3.
117. Ichiba, S., Peek, G.J., Sosnowski, A.W., Brennan, K.J., Firmin, R.K., 2000. Modifying a venovenous extracorporeal membrane oxygenation circuit to reduce recirculation. Ann Thorac Surg 69, 298–9.
118. Iglesias, M., Martinez, E., Badia, J.R., Macchiarini, P., 2008. Extrapulmonary ventilation for unresponsive severe acute respiratory distress syndrome after pulmonary resection. Ann Thorac Surg 85, 237–44; discussion 244.
119. Irby, K., Swearingen, C., Byrnes, J., Bryant, J., Prodhan, P., Fiser, R., 2012. Therapeutic heparin anti-factor XA concentrations are associated with freedom from circuit change in children supported with ECMO. Critical Care Medicine 40, 65.
120. Isgro, S., Bombino, M., Zanella, A., Milan, M., Sala, C., Foti, G., Patroniti, N., Fumagalli, R., Pesenti, A., 2010a. Low flow extracorporeal membrane oxygenation (LF-ECMO) for acute hypoxemic respiratory failure (AHRF): 10 years case-series analysis. Intensive Care Medicine 36, S357.
121. Isgro, S., Milan, M., Zanella, A., Bombino, M., Foti, G., Giani, M., Abd El Aziz El Sayed Deab, S., Patroniti, N., Pesenti, A., 2010b. Interhospital ground transportation of severe acute respiratory distress syndrome patients on extracorporeal membrane oxygenation: Monza’s experience. Critical Care 14, S69 – S70.
122. Isgro, S., Patroniti, N., Bombino, M., Marcolin, R., Zanella, A., Milan, M., Foti, G., Pesenti, A., 2011. Extracorporeal membrane oxygenation for interhospital transfer of severe acute respiratory distress syndrome patients: 5-year experience. Int J Artif Organs 34, 1052–60.
123. Javidfar, J., Brodie, D., Takayama, H., Mongero, L., Zwischenberger, J., Sonett, J., Bacchetta, M., 2011. Safe transport of critically ill adult patients on extracorporeal membrane oxygenation support to a regional extracorporeal membrane oxygenation center. ASAIO J 57, 421–5.
124. Jen, H.C., Shew, S.B., 2010. Hospital readmissions and survival after nonneonatal pediatric ECMO. Pediatrics 125, 1217–23.
125. Kalzen, H., Holzgraefe, B., Palmer, K., Frenckner, B., 2010. Right ventricular failure in patients on V-V ECMO for severe ards: Is conversion to V-A ECMO beneficial? Intensive Care Medicine 36, S359.
126. Kane, T.D., Greenhalgh, D.G., Warden, G.D., Goretsky, M.J., Ryckman, F.C., Warner, B.W., 1999. Pediatric burn patients with respiratory failure: predictors of outcome with the  use of extracorporeal life support. J Burn Care Rehabil 20, 145–50.
127. Kanji, H., Griesdale, D., Dodek, P., Reynolds, S., McCallum, J., Isac, G., Boyd, J., Gunning, D., 2012. Extracorporeal membrane oxygenation improves survival in patients with severe ARDs. Critical Care Medicine 40, 104 – 105.
128. Keyser, A., Philipp, A., Hilker, M.K., Schmid, C., 2011. Trauma patients treated with extracorporeal membrane oxygenation. Injury 42, S17 – S18.
129. Kielstein, J.T., Heiden, A.M., Beutel, G., Gottlieb, J., Wiesner, O., Hafer, C., Hadem, J., Reising, A., Haverich, A., Kuhn, C., Fischer, S., 2013. Renal function and survival in 200 patients undergoing ECMO therapy. Nephrol Dial Transplant 28, 86–90.
130. Kim, H.S., Han, S.J., Hong, K.S., Yoon, D.H., Lee, C.Y., Lee, M.-G., Hong, W.K., Lee, S.H., Kim, K.I., Lee, H.-S., Cho, S.-W., 2010. Acute respiratory failure treated with veno-venous extracorporeal membrane oxygenation. Tuberculosis and Respiratory Diseases 68, 62 – 66.
131. Kim, T.-H., Lim, C., Park, I., Kim, D.J., Jung, Y., Park, K.-H., 2012. Prognosis in the patients with prolonged extracorporeal membrane oxygenation. Korean J Thorac Cardiovasc Surg 45, 236–41.
132. Klose, A., Philipp, A., Florchinger, B., Muller, T., Bein, T., Rupprecht, L., Schmid, C., 2011. Risk of nosocomial infections in patients with acute respiratory distress syndrome (ARDS) on pumpless extracorporeal lung assist (PECLA). Thoracic and Cardiovascular Surgeon 58. P 37
133. Kolla, S., Awad, S.S., Rich, P.B., Schreiner, R.J., Hirschl, R.B., Bartlett, R.H., 1997. Extracorporeal life support for 100 adult patients with severe respiratory failure. Ann Surg 226, 544–64; discussion 565–6.
134. Kon, Z.N., Evans, C.F., Bittle, G.J., Gibber, M., Wehman, P.B., Herr, D., Rajagopal, K., Lacono, A.T., Garcia, J.P., Griffith, B.P., 2013. Outcomes of long-term veno-venous extracorporeal membrane oxygenation support for acute respiratory distress syndrome. Journal of Heart and Lung Transplantation 32, S218.
135. Kreyer, S., Muders, T., Gunther, U., Alioschat, S., Lupschen, H., Putensen, C., Wrigge, H., 2010a. Extracorporal membran oxygenation with low-dose anticoagulation. American Journal of Respiratory and Critical Care Medicine 181. S 358
136. Kreyer, S., Muders, T., Gunther, U., Alioschat, S., Lupschen, H., Putensen, C., Wrigge, H., 2010b. Extracorporal membrane oxygenation with a target-PTT of 35 sec. Intensive Care Medicine 36, S358.
137. Lamb, K., Cowan, S., Evans, N., Pitcher, H., Moritz, T., Lazar, M., Hirose, H., Cavarocchi, N., 2011. Aggressive management of bleeding complications related to extracorporeal membrane oxygenator (ECMO) resulted in successful outcomes. Chest 140. 844 A
138. Lamb, K.M., Cowan, S.W., Evans, N., Pitcher, H., Moritz, T., Lazar, M., Hirose, H., Cavarocchi, N.C., 2013. Successful management of bleeding complications in patients supported with extracorporeal membrane oxygenation with primary respiratory failure. Perfusion 28, 125–31.
139. Langham, M.R.J., Kays, D.W., Beierle, E.A., Chen, M.K., Stringfellow, K., Talbert, J.L., 2003. Expanded application of extracorporeal membrane oxygenation in a pediatric surgery practice. Ann Surg 237, 766–72; discussion 772–4.
140. Lasch, P., Varnholt, V., Kohler, B., Suske, G., Kachel, W., 1994. Extracorporeal membrane oxygenation in children with acquired respiratory distress syndrome. DAS SCHWERE ARDS IM KINDESALTER. STELLENWERT DES EXTRAKORPORALEN LUNGENERSATZES 142, 699 – 704.
141. Lazoura, O., Parthipun, A.A., Roberton, B.J., Downey, K., Finney, S., Padley, S., 2012. Acute respiratory distress syndrome related to influenza A H1N1 infection: correlation of pulmonary computed tomography findings to extracorporeal membrane  oxygenation treatment and clinical outcome. J Crit Care 27, 602–8.
142. Lebreton, G., Sanchez, B., Hennequin, J.-L., Resiere, D., Hommel, D., Leonard, C., Mehdaoui, H., Roques, F., 2012. The French airbridge for circulatory support in the Carribean. Interact Cardiovasc Thorac Surg 15, 420–5.
143. Lehle, K., Philipp, A., Gleich, O., Holzamer, A., Muller, T., Bein, T., Schmid, C., 2008. Efficiency in extracorporeal membrane oxygenation-cellular deposits on polymethylpentene membranes increase resistance to blood flow and reduce gas exchange capacity. ASAIO J 54, 612–7.
144. Lehle, K., Philipp, A., Wilm, J., Mueller, T., Lubnow, M., Schmid, C., 2011. Assessment of risk of circuit infection in patients with severe acute respiratory distress syndrome on extracorporeal life support. Thoracic and Cardiovascular Surgeon 58.
145. Lemaitre, F., Luyt, C.-E., Roullet-Renoleau, F., Nieszkowska, A., Zahr, N., Corvol, E., Fernandez, C., Antignac, M., Farinotti, R., Combes, A., 2012. Impact of extracorporeal membrane oxygenation and continuous venovenous hemodiafiltration on the pharmacokinetics of oseltamivir carboxylate in critically ill patients with pandemic (H1N1) influenza. Ther Drug Monit 34, 171–5.
146. Lewandowski, K., Rossaint, R., Pappert, D., Gerlach, H., Slama, K.-J., Weidemann, H., Frey, D.J.M., Hoffmann, O., Keske, U., Falke, K.J., 1997. High survival rate in 122 ARDS patients managed according to a clinical algorithm including extracorporeal membrane oxygenation. Intensive Care Medicine 23, 819 – 835.
147. Li, H., Meng, C., Zhu, X., Guo, L., Li, B., 2012. [The application of extracorporeal membrane oxygenation in critically ill patient]. Zhongguo Wei Zhong Bing Ji Jiu Yi Xue 24, 86–9.
148. Lidegran, M., Palmer, K., Jorulf, H., Linden, V., 2002. CT in the evaluation of patients on ECMO due to acute respiratory failure. Pediatr Radiol 32, 567–74.
149. Lidegran, M.K., Mosskin, M., Ringertz, H.G., Frenckner, B.P., Linden, V.B., 2007. Cranial CT for diagnosis of intracranial complications in adult and pediatric patients during ECMO: Clinical benefits in diagnosis and treatment. Acad Radiol 14, 62–71.
150. Lidegran, M.K., Ringertz, H.G., Frenckner, B.P., Linden, V.B., 2005. Chest and abdominal CT during extracorporeal membrane oxygenation: Clinical benefits in diagnosis and treatment. Acad Radiol 12, 276–85.
151. Lin, C.-Y., Tsai, F.-C., Tian, Y.-C., Jenq, C.-C., Chen, Y.-C., Fang, J.-T., Yang, C.-W., 2007. Evaluation of outcome scoring systems for patients on extracorporeal membrane oxygenation. Ann Thorac Surg 84, 1256–62.
152. Linden, V., Palmer, K., Reinhard, J., Westman, R., Ehren, H., Granholm, T., Frenckner, B., 2000. High survival in adult patients with acute respiratory distress syndrome treated  by extracorporeal membrane oxygenation, minimal sedation, and pressure supported  ventilation. Intensive Care Med 26, 1630–7.
153. Linden, V., Palmer, K., Reinhard, J., Westman, R., Ehren, H., Granholm, T., Frenckner, B., 2001. Inter-hospital transportation of patients with severe acute respiratory failure on extracorporeal membrane oxygenation--national and international experience. Intensive Care Med 27, 1643–8.
154. Linden, V.B., Lidegran, M.K., Frisen, G., Dahlgren, P., Frenckner, B.P., Larsen, F., 2009. ECMO in ARDS: a long-term follow-up study regarding pulmonary morphology and function and health-related quality of life. Acta Anaesthesiol Scand 53, 489–95.
155. Lindskov, C., Jensen, R.H., Sprogoe, P., Klaaborg, K.E., Kirkegaard, H., Severinsen, I.K., Lorentsen, A.G., Folkersen, L., Ilkjaer, S., Pedersen, C.M., 2013. Extracorporeal membrane oxygenation in adult patients with severe acute respiratory failure. Acta Anaesthesiol Scand 57, 303–11.
156. Lips, M., Belohlavek, J., Kunstyr, J., Mlejnsky, F., Romaniv, S., Matias, M., Kopecky, P., Lindner, J., 2010. An ECMO programme in a non-transplant cardiac centre: From postcardiotomy cardiogenic shock to swine flu. Journal of Cardiothoracic and Vascular Anesthesia 24, S47.
157. Lisboa, T., Rello, J., Moreno, R., Martin-Loeches, I., Cecconi, M., M. Pereira, J., Matos, R., Rhodes, A., 2010. Clinical characteristics in a cohort of critically ill patients infected with pandemic influenza a (H1N1)v receiving extracorporeal membrane oxigenation (ECMO) the ESICM H1N1 registry. Intensive Care Medicine 36, S371.
158. Lubnow, M., Luchner, A., Philipp, A., Buchner, S., Jeron, A., Karagiannidis, C., Bein, T., Pawlik, M., Jungbauer, C., Schmid, C., Riegger, G.A.J., Pfeifer, M., Muller, T., 2010. Combination of high frequency oscillatory ventilation and interventional lung assist in severe acute respiratory distress syndrome. J Crit Care 25, 436–44.
159. Luyt, C.-E., Combes, A., Becquemin, M.-H., Beigelman-Aubry, C., Hatem, S., Brun, A.-L., Zraik, N., Carrat, F., Grenier, P.A., Richard, J.-C.M., Mercat, A., Brochard, L., Brun-Buisson, C., Chastre, J., REVA Study Group, 2012. Long-term outcomes of pandemic 2009 influenza A(H1N1)-associated severe ARDS. Chest 142, 583–592.
160. Luyt, C.-E., Combes, A., Richard, J.-C.M., Mercat, A., Brochard, L., Brun-Buisson, C., Chastre, J., 2011. Long-termoutcomeof survivorsof acute respiratory distress syndrome (ARDS) due to pandemic 2009 influenza a (H1N1) virus infection: The respiflu study. Intensive Care Medicine 37, S287.
161. Ma, D.S., Kim, J.B., Jung, S.-H., Choo, S.J., Chung, C.H., Lee, J.W., 2012. Outcomes of venovenous extracorporeal membrane oxygenation support for acute respiratory distress syndrome in adults. Korean J Thorac Cardiovasc Surg 45, 91–4.
162. Macha, M., Griffith, B.P., Keenan, R., Kawai, A., Hattler, B.G., Fabrizio, C., Kormos, R.L., Hardesty, R.L., Boujoukos, A., Pham, S.M., 1996. ECMO support for adult patients with acute respiratory failure. ASAIO J 42, M841–4.
163. Manert, W., Haller, M., Briegel, J., Hummel, T., Kilger, E., Polasek, J., Forst, H., Peter, K., 1996. [Venovenous extracorporeal membrane oxygenation (ECMO) with a heparin-lock bypass system. An effective addition in the treatment of acute respiratory failure (ARDS)]. Anaesthesist 45, 437–48.
164. Masiakos, P.T., Islam, S., Doody, D.P., Schnitzer, J.J., Ryan, D.P., 1999. Extracorporeal membrane oxygenation for nonneonatal acute respiratory failure. Arch Surg 134, 375–9; discussion 379–80.
165. Masood, M., Jeffrey, T., Park, P., Lowell, M., Meldrum, C., Haas, C., Haft, J., Napolitano, L., 2010. Standardized ards algorithm improves safe transport of critically ill patients for ECMO evaluation at a regional ARDS center. Critical Care Medicine 38, A104.
166. Mattei, N., Mocavero, P., Corcione, A., 2011. Extracorporeal CO2 removeal in CU. European Journal of Anaesthesiology 28, 170.
167. Maul, T.M., Wolff, E.L., Kuch, B.A., Rosendorff, A., Morell, V.O., Wearden, P.D., 2012. Activated partial thromboplastin time is a better trending tool in pediatric extracorporeal membrane oxygenation. Pediatr Crit Care Med 13, e363–71.
168. Mauri, T., Bellani, G., Grasselli, G., Confalonieri, A., Rona, R., Patroniti, N., Pesenti, A., 2013. Patient-ventilator interaction in ARDS patients with extremely low compliance undergoing ECMO: a novel approach based on diaphragm electrical activity. Intensive Care Med 39, 282–91.
169. Mehta, N.M., Turner, D., Walsh, B., Zurakowski, D., Betit, P., Wilson, J., Arnold, J.H., 2010. Factors associated with survival in pediatric extracorporeal membrane oxygenation--a single-center experience. J Pediatr Surg 45, 1995–2003.
170. Mendiratta, P., Wei, J., Siraj, S., Rycus, P., Prodhan, P., 2011. Extracorporeal life support in the elderly: A report from the international elso registry. Journal of the American Geriatrics Society 59, S199.
171. Messai, E., Bouguerra, A., Harmelin, G., Di Lascio, G., Cianchi, G., Bonacchi, M., 2013. A new formula for determining arterial oxygen saturation during venovenous extracorporeal oxygenation. Intensive Care Med 39, 327–34.
172. Meyer, D.M., Jessen, M.E., 1997. Results of extracorporeal membrane oxygenation in children with sepsis. The Extracorporeal Life Support Organization. Ann Thorac Surg 63, 756–61.
173. Meyer, T.A., Warner, B.W., 1997. Extracorporeal life support for the treatment of viral pneumonia: collective experience from the ELSO registry. Extracorporeal Life Support Organization. J Pediatr Surg 32, 232–6.
174. Michaels, A.J., Hill, J.G., Long, W.B., Young, B.P., Sperley, B.P., Shanks, T.R., Morgan, L.J., 2013a. Adult refractory hypoxemic acute respiratory distress syndrome treated with extracorporeal membrane oxygenation: the role of a regional referral center. Am J Surg 205, 492–8; discussion 498–9.
175. Michaels, A.J., Hill, J.G., Long, W.B., Young, B.P., Sperley, B.P., Shanks, T.R., Morgan, L.J., 2013b. Adult refractory hypoxemic acute respiratory distress syndrome treated with extracorporeal membrane oxygenation: the role of a regional referral center. Am. J. Surg. 205, 492–498; discussion 498–499.
176. Michaels, A.J., Schriener, R.J., Kolla, S., Awad, S.S., Rich, P.B., Reickert, C., Younger, J., Hirschl, R.B., Bartlett, R.H., 1999. Extracorporeal life support in pulmonary failure after trauma. J Trauma 46, 638–45.
177. Miller, R.R., Crossno, P.F., Rice, T.W., Rodriguez, L., Shanholtz, C., Dean, N.C., Brower, R., Morris, A., 2011. Ards network registry 2009 pandemic influenza a (H1N1) infection patients with severe hypoxemia: Outcomes in those treated with and without ecmo. American Journal of Respiratory and Critical Care Medicine 183. A1638
178. Minneci, P.C., Kilbaugh, T.J., Chandler, H.K., Behar, B.J., Localio, A.R., Deans, K.J., 2013. Factors associated with mortality in pediatric patients requiring extracorporeal  life support for severe pneumonia. Pediatr Crit Care Med 14, e26–33.
179. Moler, F.W., Custer, J.R., Bartlett, R.H., Palmisano, J.M., Akingbola, O., Taylor, R.P., Maxvold, N.J., 1994a. Extracorporeal life support for severe pediatric respiratory failure: an updated  experience 1991-1993. J Pediatr 124, 875–80.
180. Moler, F.W., Palmisano, J.M., Custer, J.R., Bartlett, R.H., 1996. Mechanical ventilation and arterial blood gas measurements 24 hours postextracorporeal life support for survivors of pediatric respiratory failure. Crit Care Med 24, 679–82.
181. Moler, F.W., Palmisano, J.M., Custer, J.R., Meliones, J.N., Bartlett, R.H., 1994b. Alveolar-arterial oxygen gradients before extracorporeal life support for severe  pediatric respiratory failure: improved outcome for extracorporeal life support-managed patients? Crit Care Med 22, 620–5.
182. Mols, G., Loop, T., Geiger, K., Farthmann, E., Benzing, A., 2000. Extracorporeal membrane oxygenation: a ten-year experience. Am J Surg 180, 144–54.
183. Mols, G., Loop, T., Hermle, G., Buttler, J., Huber, B., Schubert, J., Benzing, A., 2001. [10 years experience with extracorporeal membrane oxygenation]. Anasthesiol Intensivmed Notfallmed Schmerzther 36, 4–14.
184. Moreno Franco, P., Rachmale, S., Ding, S., Wilson, G., Li, G., 2011. Rescue therapies in patients with refractory hypoxemia. American Journal of Respiratory and Critical Care Medicine 183. A 1643
185. Morris, A.H., Wallace, C.J., Menlove, R.L., Clemmer, T.P., Orme Jr., J.F., Weaver, L.K., Dean, N.C., Thomas, F., East, T.D., Pace, N.L., Suchyta, M.R., Beck, E., Bombino, M., Sittig, D.F., Bohm, S., Hoffmann, B., Becks, H., Butler, S., Pearl, J., Rasmusson, B., 1994. Randomized clinical trial of pressure-controlled inverse ratio ventilation and extracorporeal CO2 removal for Adult Respiratory Distress Syndrome. American Journal of Respiratory and Critical Care Medicine 149, 295 – 305.
186. Morton, A., Dalton, H., Kochanek, P., Janosky, J., Thompson, A., 1994. Extracorporeal membrane oxygenation for pediatric respiratory failure: five-year  experience at the University of Pittsburgh. Crit Care Med 22, 1659–67.
187. Muellenbach, R.M., Kredel, M., Wunder, C., Kustermann, J., Wurmb, T., Schwemmer, U., Schuster, F., Anetseder, M., Roewer, N., Brederlau, J., 2008. Arteriovenous extracorporeal lung assist as integral part of a multimodal treatment concept: a retrospective analysis of 22 patients with ARDS refractory to standard care. Eur J Anaesthesiol 25, 897–904.
188. Muller, T., Philipp, A., Luchner, A., Karagiannidis, C., Bein, T., Hilker, M., Rupprecht, L., Langgartner, J., Zimmermann, M., Arlt, M., Wenger, J., Schmid, C., Riegger, G.A., Pfeifer, M., Lubnow, M., 2009. A new miniaturized system for extracorporeal membrane oxygenation in adult respiratory failure. Crit Care 13, R205.
189. Murala, J.S., Swain, S.K., Kavunkal, A.M., Singappuli, K., Karl, T.R., Nunn, G.R., Provenzano, S.C., 2010. Initial experience with the Jostra-Quadrox extracorporeal life support system in a new pediatric cardiac unit. Annals of Pediatric Cardiology 3, 218.
190. Muralidharan, R., Mateen, F., Schears, G., Wijdicks, E., 2010. The challenges of brain death determination in patients on extracorporeal membrane xygenation (ECMO). Neurocritical Care 13, S37.
191. Nair, P., Davies, A.R., Beca, J., Bellomo, R., Ellwood, D., Forrest, P., Jackson, A., Pye, R., Seppelt, I., Sullivan, E., Webb, S., 2011. Extracorporeal membrane oxygenation for severe ARDS in pregnant and postpartum women during the 2009 H1N1 pandemic. Intensive Care Med 37, 648–54.
192. Nance, M.L., Nadkarni, V.M., Hedrick, H.L., Cullen, J.A., Wiebe, D.J., 2009. Effect of preextracorporeal membrane oxygenation ventilation days and age on extracorporeal membrane oxygenation survival in critically ill children. J Pediatr Surg 44, 1606–10.
193. Nehra, D., Goldstein, A.M., Doody, D.P., Ryan, D.P., Chang, Y., Masiakos, P.T., 2009. Extracorporeal membrane oxygenation for nonneonatal acute respiratory failure: the Massachusetts General Hospital experience from 1990 to 2008. Arch Surg 144, 427–32; discussion 432.
194. Nicolas, J.-B., Cambier, J.-F., Bouhon, S., Bulpa, P., Gonzalez, M., Michaux, I., Installe, E., Dive, A., Evrard, P., 2010. Is mobile extracorporeal membrane oxygenation a safe tool to transfer severely hypoxaemic patients in case of influenza a/H1N1 infection? Intensive Care Medicine 36, S236.
195. Niederbichler, A.D., Jokuszies, A., Peters, T., Steiert, A., Knobloch, K., Busch, K., Vogt Plastic, P., 2009. Extracorporeal life support devices (ECMO, ILA) in severely burned patients: Bridging the gap? Burns 35, S45.
196. Nierhaus, A., Frings, D., Braune, S., Baumann, H.-J., Schneider, C., Wittenburg, B., Kluge, S., 2011. Interventional lung assist enables lung protective mechanical ventilation in acute respiratory distress syndrome. Minerva Anestesiol 77, 797–801.
197. Nitsch, S., Kalenka, A., 2010. Acute respiratory distress syndrome on the basis of 2009 novel swine-origin influenza a (H1N1) infection: Report from an ecmo-center in germany. Intensive Care Medicine 36, S235.
198. Nitsch, S., Kalenka, A., 2011. Extracorporeal life support for acute respiratory distress syndrome: Using a mobile ECMO system and low-dose anticoagulation. Intensive Care Medicine 37, S210.
199. Noah, M.A., Peek, G.J., Finney, S.J., Griffiths, M.J., Harrison, D.A., Grieve, R., Sadique, M.Z., Sekhon, J.S., McAuley, D.F., Firmin, R.K., Harvey, C., Cordingley, J.J., Price, S., Vuylsteke, A., Jenkins, D.P., Noble, D.W., Bloomfield, R., Walsh, T.S., Perkins, G.D., Menon, D., Taylor, B.L., Rowan, K.M., 2011. Referral to an extracorporeal membrane oxygenation center and mortality among patients with severe 2009 influenza A(H1N1). JAMA 306, 1659–68.
200. Noah, M.A., Ramachandra, G., Hickey, M.M., Jenkins, D.R., Harvey, C.J., Westrope, C.A., Firmin, R.K., Peek, G.J., 2013. Extracorporeal membrane oxygenation and severe acute respiratory distress secondary to legionella: 10 year experience. ASAIO J 59, 328–30.
201. Norfolk, S.G., Hollingsworth, C.L., Wolfe, C.R., Govert, J.A., Que, L.G., Cheifetz, I.M., Hollingsworth, J.W., 2010. Rescue therapy in adult and pediatric patients with pH1N1 influenza infection: a  tertiary center intensive care unit experience from April to October 2009. Crit Care Med 38, 2103–7.
202. Nunes, A.J., Hruczkowski, J., MacArthur, R.G., 2012. Edmonton experience with venovenous extracorporeal membrane oxygenation in critically ill adults with severe respiratory failure. Canadian Journal of Cardiology 28, S399.
203. O’Brien, N., 2011. Extra corporeal membrane oxygenation (ECMO) and cerebral blood flow velocity (CBFV) in children. Critical Care Medicine 39, 7.
204. Oshima, K., Kunimoto, F., Hinohara, H., Ohkawa, M., Mita, N., Tajima, Y., Saito, S., 2010a. Extracorporeal membrane oxygenation for respiratory failure: comparison of venovenous versus venoarterial bypass. Surg Today 40, 216–22.
205. Oshima, K., Kunimoto, F., Hinohara, H., Okawa, M., Mita, N., Kanemaru, Y., Tajima, Y., Saito, S., 2010b. Evaluation of prognosis in patients with respiratory failure requiring venovenous extracorporeal membrane oxygenation (ECMO). Ann Thorac Cardiovasc Surg 16, 156–62.
206. Paden, M.L., Conrad, S.A., Rycus, P.T., Thiagarajan, R.R., 2013. Extracorporeal life support organization registry report 2012. ASAIO J 59, 202–10.
207. Panarello, G., Occhipinti, G., Capitanio, G., Ferrazza, V., Vitulo, P., Pilato, M., Di Lorenzo, G., Arcadipane, A.F., 2010. Treatment of a/H1N1 related severe ards by extracorporeal membrane oxygenation in pregnant and postpartum women, during the 2009 flu pandemic. Single center experience. Intensive Care Medicine 36, S235.
208. Panigada, M., Mietto, C., Pagan, F., Bogno, L., Berto, V., Gattinoni, L., 2013. Monitoring anticoagulation during extracorporeal membrane oxygenation in patients with acute respiratory failure. Critical Care 17, S47 – S48.
209. Papadopoulos, N., Ahmad, A.E.-S., Marinos, S., Moritz, A., Zierer, A., 2012. Extracorporeal Membrane Oxygenation for Influenza-Associated Acute Respiratory Distress Syndrome. Thorac Cardiovasc Surg. 516 - 21
210. Pappalardo, F., Pieri, M., Greco, T., Patroniti, N., Pesenti, A., Arcadipane, A., Ranieri, V.M., Gattinoni, L., Landoni, G., Holzgraefe, B., Beutel, G., Zangrillo, A., 2013. Predicting mortality risk in patients undergoing venovenous ECMO for ARDS due to  influenza A (H1N1) pneumonia: the ECMOnet score. Intensive Care Med 39, 275–81.
211. Park, P., Rycus, P., Bembea, M., Oldenburg, G., Annich, G., Haft, J., Blum, J., Napolitano, L., Bartlett, R., 2009. Influenza and ECMO for severe respiratory failure: Review of the 20-year ELSO experience. Critical Care Medicine 37, A338.
212. Park, Y., Hwang, S., Lee, S.G., 2012. Intensive pulmonary support using extracorporeal membrane oxygenation for adult liver transplant recipients with respiratory failure. American Journal of Transplantation 12, 439.
213. Park, Y.-H., Hwang, S., Park, H.-W., Park, C.-S., Lee, H.-J., Namgoong, J.-M., Yoon, S.-Y., Jung, S.-W., Song, G.-W., Park, G.-C., Jung, D.-H., Ahn, C.-S., Kim, K.-H., Moon, D.-B., Ha, T.-Y., Lee, S.-G., 2012. Effect of pulmonary support using extracorporeal membrane oxygenation for adult liver transplant recipients with respiratory failure. Transplant Proc 44, 757–61.
214. Pasquini, A., Di Valvasone, S., Biondi, S., Batacchi, S., Cianchi, G., Ciapetti, M., Bonizzoli, M., Spina, R., Turrisi, L., Mascitelli, E., Bonacchi, M., Zagli, G., Gensini, G.F., Peris, A., 2010. Extracorporeal membrane oxygenation for influenza A (H1N1): Experience in a regional referral center. Critical Care 14, S32 – S33.
215. Pathan, N., Ridout, D.A., Smith, E., Goldman, A.P., Brown, K.L., 2008. Predictors of outcome for children requiring respiratory extra-corporeal life support: implications for inclusion and exclusion criteria. Intensive Care Med 34, 2256–63.
216. Patroniti, N., Zangrillo, A., Pappalardo, F., Peris, A., Cianchi, G., Braschi, A., Iotti, G.A., Arcadipane, A., Panarello, G., Ranieri, V.M., Terragni, P., Antonelli, M., Gattinoni, L., Oleari, F., Pesenti, A., 2011. The Italian ECMO network experience during the 2009 influenza A(H1N1) pandemic: preparation for severe respiratory emergency outbreaks. Intensive Care Med 37, 1447–57.
217. Peek, G.J., Clemens, F., Elbourne, D., Firmin, R., Hardy, P., Hibbert, C., Killer, H., Mugford, M., Thalanany, M., Tiruvoipati, R., Truesdale, A., Wilson, A., 2006. CESAR: Conventional ventilatory support vs extracorporeal membrane oxygenation for severe adult respiratory failure. BMC Health Services Research 6: 163
218. Peek, G.J., Elbourne, D., Mugford, M., Tiruvoipati, R., Wilson, A., Allen, E., Clemens, F., Firmin, R., Hardy, P., Hibbert, C., Jones, N., Killer, H., Thalanany, M., Truesdale, A., 2010. Randomised controlled trial and parallel economic evaluation of conventional ventilatory support versus extracorporeal membrane oxygenation for severe adult respiratory failure (CESAR). Health Technology Assessment 14, 1 – 73.
219. Peek, G.J., Moore, H.M., Moore, N., Sosnowski, A.W., Firmin, R.K., 1997. Extracorporeal membrane oxygenation for adult respiratory failure. Chest 112, 759–64.
220. Peek, G.J., Mugford, M., Tiruvoipati, R., Wilson, A., Allen, E., Thalanany, M.M., Hibbert, C.L., Truesdale, A., Clemens, F., Cooper, N., Firmin, R.K., Elbourne, D., 2009. Efficacy and economic assessment of conventional ventilatory support versus extracorporeal membrane oxygenation for severe adult respiratory failure (CESAR): a multicentre randomised controlled trial. Lancet 374, 1351–63.
221. Peng, C.-C., Wu, S.-J., Chen, M.-R., Chiu, N.-C., Chi, H., 2012. Clinical experience of extracorporeal membrane oxygenation for acute respiratory  distress syndrome associated with pneumonia in children. J Formos Med Assoc 111, 147–52.
222. Peris, A., Cianchi, G., Biondi, S., Bonizzoli, M., Pasquini, A., Bonacchi, M., Ciapetti, M., Zagli, G., Bacci, S., Lazzeri, C., Bernardo, P., Mascitelli, E., Sani, G., Gensini, G.F., 2010. Extracorporeal life support for management of refractory cardiac or respiratory failure: initial experience in a tertiary centre. Scand J Trauma Resusc Emerg Med 18, 28.
223. Pettignano, R., Fortenberry, J.D., Heard, M.L., Labuz, M.D., Kesser, K.C., Tanner, A.J., Wagoner, S.F., Heggen, J., 2003. Primary use of the venovenous approach for extracorporeal membrane oxygenation in pediatric acute respiratory failure. Pediatr Crit Care Med 4, 291–8.
224. Pham, T., Combes, A., Roze, H., Chevret, S., Mercat, A., Roch, A., Mourvillier, B., Ara-Somohano, C., Bastien, O., Zogheib, E., Clavel, M., Constan, A., Marie Richard, J.-C., Brun-Buisson, C., Brochard, L., 2013. Extracorporeal membrane oxygenation for pandemic influenza A(H1N1)-induced acute  respiratory distress syndrome: a cohort study and propensity-matched analysis. Am J Respir Crit Care Med 187, 276–85.
225. Philipp, A., Arlt, M., Zimmermann, M., Foltan, M., Bein, T., Muller, T., Rupprecht, L., Hilker, M., Schmid, C., 2011. Interhospital transfer with extracorporeal cardiopulmonary support in critically ill patients. Thoracic and Cardiovascular Surgeon 58. P 30
226. Pranikoff, T., Hirschl, R.B., Remenapp, R., Swaniker, F., Bartlett, R.H., 1999. Venovenous extracorporeal life support via percutaneous cannulation in 94 patients. Chest 115, 818–22.
227. Pranikoff, T., Hirschl, R.B., Steimle, C.N., Anderson, H.L. 3rd, Bartlett, R.H., 1994. Efficacy of extracorporeal life support in the setting of adult cardiorespiratory failure. ASAIO J 40, M339–43.
228. Pranikoff, T., Hirschl, R.B., Steimle, C.N., Anderson, H.L. 3rd, Bartlett, R.H., 1997. Mortality is directly related to the duration of mechanical ventilation before the initiation of extracorporeal life support for severe respiratory failure. Crit Care Med 25, 28–32.
229. Rachmale, S., Li, G., Ahmed, A., Kashyap, R., Bice, T., Wilson, M., Gajic, O., 2010. Use of rescue therapies for ards patients: Population-based study in olmstead county, Minnesota. Critical Care Medicine 38, A103.
230. Redaelli, S., Milan, M., Zanella, A., Isgro, S., Magni, F., Patroniti, N., Pesenti, A., 2010. Daily nursing in patients during venous-venous ECMO: Observational study. Intensive Care Medicine 36, S356.
231. Reeb, J., Falcoz, P.E., Pottecher, J., Delabranche, X., Santelmo, N., Steib, A., Hasselmann, M., Massard, G., 2012. Two years’ experience with bicaval dual lumen cannula for venovenous extracorporeal membrane oxygenation in adult refractory acute respiratory distress syndrome. Critical Care 16, S36.
232. Reed, R.C., Rutledge, J.C., 2009. Thrombotic and hemorrhagic complications in pediatric extracorporeal membrane oxygenation (ECMO) nonsurvivors: An autopsy series. Pediatric and Developmental Pathology 12, 313 – 314.
233. Reed, R.C., Rutledge, J.C., 2010. Laboratory and clinical predictors of thrombosis and hemorrhage in 29 pediatric extracorporeal membrane oxygenation nonsurvivors. Pediatr Dev Pathol 13, 385–92.
234. Rega, F.R., Evrard, V., Bollen, H., Peeters, G., Vercaemst, L., Meuris, B., Herijgers, P., Sergeant, P., Hermans, G., Vlasselaers, D., Meyns, B., 2007. pH 48 h after onset of extracorporeal membrane oxygenation is an independent predictor of survival in patients with respiratory failure. Artif Organs 31, 384–9.
235. Rehder, K.J., Turner, D.A., Cheifetz, I.M., 2013. Extracorporeal membrane oxygenation for neonatal and pediatric respiratory failure: an evidence-based review of the past decade (2002-2012). Pediatr Crit Care Med 14, 851–861.
236. Rich, P.B., Awad, S.S., Crotti, S., Hirschl, R.B., Bartlett, R.H., Schreiner, R.J., 1998a. A prospective comparison of atrio-femoral and femoro-atrial flow in adult venovenous extracorporeal life support. J Thorac Cardiovasc Surg 116, 628–32.
237. Rich, P.B., Awad, S.S., Kolla, S., Annich, G., Schreiner, R.J., Hirschl, R.B., Bartlett, R.H., 1998b. An approach to the treatment of severe adult respiratory failure. J Crit Care 13, 26–36.
238. Rich, P.B., Younger, J.G., Soldes, O.S., Awad, S.S., Bartlett, R.H., 1998c. Use of extracorporeal life support for adult patients with respiratory failure and sepsis. ASAIO J 44, 263–6.
239. Risnes, I., Wagner, K., Nome, T., Sundet, K., Jensen, J., Hynas, I.A., Ueland, T., Pedersen, T., Svennevig, J.L., 2006. Cerebral outcome in adult patients treated with extracorporeal membrane oxygenation. Annals of Thoracic Surgery 81, 1401 – 1406.
240. Roch, A., Hraiech, S., Masson, E., Grisoli, D., Forel, J.-M., Boucekine, M., Morera, P., Guervilly, C., Adda, M., Dizier, S., Toesca, R., Collart, F., Papazian, L., 2014. Outcome of acute respiratory distress syndrome patients treated with extracorporeal membrane oxygenation and brought to a referral center. Intensive Care Med 40, 74–83.
241. Roch, A., Lepaul-Ercole, R., Grisoli, D., Bessereau, J., Brissy, O., Castanier, M., Dizier, S., Forel, J.-M., Guervilly, C., Gariboldi, V., Collart, F., Michelet, P., Perrin, G., Charrel, R., Papazian, L., 2010. Extracorporeal membrane oxygenation for severe influenza A (H1N1) acute respiratory distress syndrome: a prospective observational comparative study. Intensive Care Med 36, 1899–905.
242. Roch, A., Ollier, S., Guervilly, C., Forel, J.-M., Adda, M., Hraiech, S., Xeridat, F., Papazian, L., 2012. Prognostic factors during extracorporeal membrane oxygenation for ARDS: Interest of the sequential organ failure (SOFA) score. Intensive Care Medicine 38, S259.
243. Roger, D., Dudouit, J.-M., Résière, D., Mehdaoui, H., Courcier, D., Villain, L., Léonard, C., Roques, F., Lebreton, G., 2013b. [Interhospital transfer of ECMO-assisted patients in Martinique]. Ann Fr Anesth Reanim 32, 307–314.
244. Rollins, M.D., Hubbard, A., Zabrocki, L., Barnhart, D.C., Bratton, S.L., 2012. Extracorporeal membrane oxygenation cannulation trends for pediatric respiratory  failure and central nervous system injury. J Pediatr Surg 47, 68–75.
245. Roncon-Albuquerque, R.J., Basilio, C., Figueiredo, P., Silva, S., Mergulhao, P., Alves, C., Veiga, R., Castelo-Branco, S., Paiva, L., Santos, L., Honrado, T., Dias, C., Oliveira, T., Sarmento, A., Mota, A.M., Paiva, J.A., 2012. Portable miniaturized extracorporeal membrane oxygenation systems for H1N1-related severe acute respiratory distress syndrome: a case series. J Crit Care 27, 454–63.
246. Rossaint, R., Pappert, D., Gerlach, H., Lewandowski, K., Keh, D., Falke, K., 1997. Extracorporeal membrane oxygenation for transport of hypoxaemic patients with severe ARDS. Br J Anaesth 78, 241–6.
247. Roussel, A., Al-Attar, N., Alkhoder, S., Radu, C., Raffoul, R., Alshammari, M., Montravers, P., Wolff, M., Nataf, P., 2012. Outcomes of percutaneous femoral cannulation for venoarterial extracorporeal membrane oxygenation support. European Heart Journal: Acute Cardiovascular Care 1, 111 – 114.
248. Rulisek, J., Leden, P., Zakharchenko, M., Bartakova, H., Belohlavek, J., Kunstyr, J., Balik, M., 2012. Can initial inflammatory and coagulation parameters predict duration and outcome of VV-ECMO therapy in adults? Intensive Care Medicine 38, S259.
249. Saeed, D., Islamovic, M., Mysliwiec, H., Kamiya, H., Maxhera, B., Albert, A., Lichtenberg, A., 2011. Neurological outcome in patients receiving veno-venous versus veno-arterial extracorporeal membrane oxygenation. Heart Surgery Forum 14, S108 – S109.
250. Sagy, M., Esperanza, M.C., Silver, P.C., 2008. Factors influencing outcome of patients receiving extracorporeal membrane oxygenation for neonatal and pediatric respiratory failure. Current Respiratory Medicine Reviews 4, 69 – 76.
251. Schellongowski, P., Ullrich, R., Hieber, C., Hetz, H., Losert, H., Hermann, M., Hermann, A., Gattringer, K.-B., Siersch, V., Rabitsch, W., Fuhrmann, V., Bojic, A., Robak, O., Sperr, W.R., Laczika, K., Locker, G.J., Staudinger, T., 2011. A surge of flu-associated adult respiratory distress syndrome in an Austrian tertiary care hospital during the 2009/2010 Influenza A H1N1v pandemic. Wien Klin Wochenschr 123, 209–14.
252. Schellongowski, P., Ullrich, R., Robak, O., Bojic, A., Hermann, A., Sperr, W.R., Rabitsch, W., Knoebl, P., Fuhrmann, V., Laczika, K., Locker, G.J., Staudinger, T., 2012. Extracorporeal membrane oxygenation in patients with hematologic malignancies and severe acute respiratory distress syndrome. Intensive Care Medicine 38, S164.
253. Schmidt, M., Tachon, G., Devilliers, C., Muller, G., Hekimian, G., Brechot, N., Merceron, S., Luyt, C.E., Trouillet, J.-L., Chastre, J., Leprince, P., Combes, A., 2013a. Blood oxygenation and decarboxylation determinants during venovenous ECMO for respiratory failure in adults. Intensive Care Med 39, 838–46.
254. Schmidt, M., Zogheib, E., Rozé, H., Repesse, X., Lebreton, G., Luyt, C.-E., Trouillet, J.-L., Bréchot, N., Nieszkowska, A., Dupont, H., Ouattara, A., Leprince, P., Chastre, J., Combes, A., 2013b. The PRESERVE mortality risk score and analysis of long-term outcomes after extracorporeal membrane oxygenation for severe acute respiratory distress syndrome. Intensive Care Med 39, 1704–1713.
255. Scott, L.K., Boudreaux, K., Thaljeh, F., Grier, L.R., Conrad, S.A., 2004. Early enteral feedings in adults receiving venovenous extracorporeal membrane oxygenation. JPEN J Parenter Enteral Nutr 28, 295–300.
256. Sedgwick, J.F., Burstow, D.J., Platts, D.G., 2011. The role of echocardiography in the management of patients supported by extracorporeal membranous oxygenation (ECMO). International Journal of Cardiology 147, S16 – S17.
257. Segura, S., Cambra, F.J., Moreno, J., Thio, M., Riverola, A., Iriondo, M., Mayol, J., Palomeque, A., 2009. [ECMO: experience in paediatrics]. An Pediatr (Barc) 70, 12–9.
258. Serra De Oliveira, B., Mendes Fernandes, S., Francnulla, C., 2013. Nosocomial bloodstream infection and venovenous extracorporeal membrane oxygenation: A retrospective cohort study. Critical Care 17, S47.
259. Shafii, A.E., Brown, C.R., Murthy, S.C., Mason, D.P., 2012. High incidence of upper-extremity deep vein thrombosis with dual-lumen venovenous extracorporeal membrane oxygenation. Journal of Thoracic and Cardiovascular Surgery 144, 988 – 989.
260. Shekar, K., Roberts, J.A., Mullany, D.V., Corley, A., Fisquet, S., Bull, T.N., Barnett, A.G., Fraser, J.F., 2012. Increased sedation requirements in patients receiving extracorporeal membrane oxygenation for respiratory and cardiorespiratory failure. Anaesth Intensive Care 40, 648–55.
261. Shekar, K., Roberts, J.A., Welch, S., Buscher, H., Rudham, S., Burrows, F., Ghassabian, S., Wallis, S.C., Levkovich, B., Pellegrino, V., McGuinness, S., Parke, R., Gilder, E., Barnett, A.G., Walsham, J., Mullany, D.V., Fung, Y.L., Smith, M.T., Fraser, J.F., 2012. ASAP ECMO: Antibiotic, Sedative and Analgesic Pharmacokinetics during Extracorporeal Membrane Oxygenation: a multi-centre study to optimise drug therapy during ECMO. BMC Anesthesiol 12, 29.
262. Solaro, M., Bambi, S., Bacchi, I., Bendoni, E., Dammiano, P., Trevisan, M., Migliaccio, M.L., Cecchi, A., Cammelli, R., Bonizzoli, M., Peris, A., 2011. Quality of life in H1N1-induced ards survivors: Data from multidisciplinary follow up clinics in a referral ECMO center. Intensive Care Medicine 37, S174.
263. Starck, C.T., Hasenclever, P., Falk, V., Wilhelm, M.J., 2013. Interhospital transfer of seriously sick ARDS patients using veno-venous Extracorporeal Membrane Oxygenation (ECMO): Concept of an ECMO transport team. Int J Crit Illn Inj Sci 3, 46–50.
264. Stewart, D.L., Dela Cruz, T.V., Ziegler, C., Goldsmith, L.J., 1997. The use of extracorporeal membrane oxygenation in patients with gram-negative or  viral sepsis. Perfusion 12, 3–8.
265. Stohr, F., Emmert, M.Y., Lachat, M.L., Stocker, R., Maggiorini, M., Falk, V., Wilhelm, M.J., 2011. Extracorporeal membrane oxygenation for acute respiratory distress syndrome: is the configuration mode an important predictor for the outcome? Interact Cardiovasc Thorac Surg 12, 676–80.
266. Sun, B., Wang, C., Wu, J., Li, X., He, H., Zhang, C., Tong, Z., Zhan, Q., Wang, C., 2012. [The experience of extracorporeal membrane oxygenation for severe acute respiratory failure in adults]. Zhonghua Jie He He Hu Xi Za Zhi 35, 804–8.
267. Swaniker, F., Kolla, S., Moler, F., Custer, J., Grams, R., Barlett, R., Hirschl, R., 2000. Extracorporeal life support outcome for 128 pediatric patients with respiratory failure. J Pediatr Surg 35, 197–202.
268. Swol, J., Buchwald, D., Schildhauer, T.A., 2012. Use of VENO-venous extracorporeal membrane oxygenation (V.-V. ECMO) to ensure the lung protective ventilation in trauma patients. Intensive Care Medicine 38, S261 –.
269. Takeda, S., Kotani, T., Nakagawa, S., Ichiba, S., Aokage, T., Ochiai, R., Taenaka, N., Kawamae, K., Nishimura, M., Ujike, Y., Tajimi, K., 2012. Extracorporeal membrane oxygenation for 2009 influenza A(H1N1) severe respiratory failure in Japan. J Anesth 26, 650–7.
270. Tao, L., Zhang, J., Ai, H., Zhao, S., Cai, H., Ning, J., Xu, H., 2004. [Extracorporeal membrane oxygenation treatment with high-volume hemofiltration in patients with multiple organ dysfunction syndrome]. Zhongguo Wei Zhong Bing Ji Jiu Yi Xue 16, 723–6.
271. Terragni, P.P., Del Sorbo, L., Mascia, L., Urbino, R., Martin, E.L., Birocco, A., Faggiano, C., Quintel, M., Gattinoni, L., Ranieri, V.M., 2009. Tidal volume lower than 6 ml/kg enhances lung protection: role of extracorporeal  carbon dioxide removal. Anesthesiology 111, 826–35.
272. Touloukian, R.J., 2009. Extracorporeal membrane oxygenation for nonneonatal acute respiratory failure: The Massachusetts General Hospital experience from 1990 to 2008 - Invited critique. Archives of Surgery 144, 432.
273. Tseng, Y.-H., Wu, M.-Y., Tsai, F.-C., Chen, H.-J., Lin, P.J., 2011. Costs associated with extracorporeal life support used in adults: A single-center study. Acta Cardiologica Sinica 27, 221 – 228.
274. Turner, D.A., Rehder, K.J., Peterson-Carmichael, S.L., Ozment, C.P., Al-Hegelan, M.S., Williford, W.L., Peters, M.A., Noble, P.W., Cheifetz, I.M., 2011. Extracorporeal membrane oxygenation for severe refractory respiratory failure secondary to 2009 H1N1 influenza A. Respir Care 56, 941–6.
275. Ullrich, R., Lorber, C., Roder, G., Urak, G., Faryniak, B., Sladen, R.N., Germann, P., 1999. Controlled airway pressure therapy, nitric oxide inhalation, prone position, and  extracorporeal membrane oxygenation (ECMO) as components of an integrated approach to ARDS. Anesthesiology 91, 1577–86.
276. Vats, A., Pettignano, R., Culler, S., Wright, J., 1998. Cost of extracorporeal life support in pediatric patients with acute respiratory  failure. Crit Care Med 26, 1587–92.
277. Weber, T.R., Kountzman, B., 1998. Extracorporeal membrane oxygenation for nonneonatal pulmonary and multiple-organ  failure. J Pediatr Surg 33, 1605–9.
278. Weber-Carstens, S., Bercker, S., Hommel, M., Deja, M., MacGuill, M., Dreykluft, C., Kaisers, U., 2009. Hypercapnia in late-phase ALI/ARDS: providing spontaneous breathing using pumpless extracorporeal lung assist. Intensive Care Med 35, 1100–5.
279. Wilhelm, M.J., Sahin, A., Staab, R., Hasenclever, P., Falk, V., 2011a. Emergency ECMO implantation at peripheral hospitals with subsequent patient transport to the tertiary care center. Thoracic and Cardiovascular Surgeon 59. eP66
280. Wilhelm, M.J., Stohr, F., Emmert, M., Lachat, M.L., Falk, V., 2011b. Extracorporeal membrane oxygenation for ARDS: Aspects of cannulation mode. Thoracic and Cardiovascular Surgeon 59. eP69
281. Wong, J.K., Pitcher, H., Hirose, H., De Caro, M., Cavarocchi, N.C., 2012. End-organ recovery and survival with the quadroxd oxygenator in adults on ECMO. Artificial Organs 36, A40 – A41.
282. Wong, T.E., Delaney, M., Gernsheimer, T.B., Matthews, D.C., Brogan, T., Mazor, R., McMullan, D.M., Reiner, A.P., Konkle, B.A., 2012. Antithrombin concentrate use in pediatric extracorporeal membrane oxygenation: A retrospective cohort study. Blood 120. 1170
283. Wu, S.-J., 2011. Extracorporeal membrane oxygenation for acute respiratory distress syndrome associated with pneumonia in children. Artificial Organs 35, A14.
284. Wu, S.-J., Chen, M.-R., Sun, S., Li, J.-Y., 2007. Extra-corporeal membrane oxygenation for acute respiratory distress syndrome: A single center experience. Acta Cardiologica Sinica 23, 97 – 102.
285. Wu, V.-C., Tsai, H.-B., Yeh, Y.-C., Huang, T.-M., Lin, Y.-F., Chou, N.-K., Chen, Y.-S., Han, Y.-Y., Chou, A., Lin, Y.-H., Wu, M.-S., Lin, S.-L., Chen, Y.-M., Tsai, P.-R., Ko, W.-J., Wu, K.-D., 2010. Patients supported by extracorporeal membrane oxygenation and acute dialysis: Acute physiology and chronic health evaluation score in predicting hospital mortality. Artificial Organs 34, 828 – 835.
286. Yamagishi, T., Ishikawa, S., Otaki, A., Otani, Y., Takahashi, T., Sato, Y., Yoshida, I., Kunimoto, F., Arai, K., Morishita, Y., 1995. Extracorporeal membrane oxygenation for respiratory failure. Surg Today 25, 690–3.
287. Yamagishi, T., Kunimoto, F., Isa, Y., Hinohara, H., Morishita, Y., 2004. Clinical results of extracorporeal membrane oxygenation (ECMO) support for acute  respiratory failure: a comparison of a centrifugal pump ECMO with a roller pump ECMO. Surg Today 34, 209–13.
288. Yan, J., Zhou, M., Zhu, Y., Xu, H., Yang, T., Chen, J., 2012. [Extracorporeal membrane oxygenation for acute respiratory distress syndrome: a study of 11 cases]. Zhonghua Jie He He Hu Xi Za Zhi 35, 809–13.
289. Zabrocki, L.A., Brogan, T.V., Statler, K.D., Poss, W.B., Rollins, M.D., Bratton, S.L., 2011. Extracorporeal membrane oxygenation for pediatric respiratory failure: Survival and predictors of mortality. Crit Care Med 39, 364–70.
290. Zahraa, J.N., Moler, F.W., Annich, G.M., Maxvold, N.J., Bartlett, R.H., Custer, J.R., 2000. Venovenous versus venoarterial extracorporeal life support for pediatric respiratory failure: are there differences in survival and acute complications? Crit Care Med 28, 521–5.
291. Zamora, I.J., Shekerdemian, L., Olutoye, O.O., Cass, D.L., Rycus, P., Burgman, C., Lee, T.C., 2013. An analysis of outcomes comparing dual-lumen venovenous ECMO to multi-site venovenous ECMO for pediatric respiratory failure: The extracorporeal life support registry experience. Journal of Surgical Research 179, 195
292. Zampieri, F.G., Mendes, P.V., Ranzani, O.T., Taniguchi, L.U., Pontes Azevedo, L.C., Vieira Costa, E.L., Park, M., 2013. Extracorporeal membrane oxygenation for severe respiratory failure in adult patients: a systematic review and meta-analysis of current evidence. J Crit Care 28, 998–1005.
293. Zapol, W.M., Snider, M.T., Hill, J.D., 1979. Extracorporeal membrane oxygenation in severe acute respiratory failure. A randomized prospective study. Journal of the American Medical Association 242, 2193 – 2196.
294. Zhan, Q., Sun, B., Tong, Z., Guo, L., Xu, L., Zhu, X., Jia, X., Wang, C., 2011. [Extracorporeal membrane oxygenation for severe acute respiratory distress syndrome caused by novel 2009 influenza A (H1N1) virus]. Zhonghua Yi Xue Za Zhi 91, 3262–6.
295. Zimmermann, M., Bein, T., Arlt, M., Philipp, A., Rupprecht, L., Mueller, T., Lubnow, M., Graf, B.M., Schlitt, H.J., 2009. Pumpless extracorporeal interventional lung assist in patients with acute respiratory distress syndrome: a prospective pilot study. Crit Care 13, R10.
296. Zimmermann, M., Bein, T., Philipp, A., Ittner, K., Foltan, M., Drescher, J., Weber, F., Schmid, F.-X., 2006. Interhospital transportation of patients with severe lung failure on pumpless extracorporeal lung assist. Br J Anaesth 96, 63–6.
297. Zogheib, E., Buchalet, C., Guinot, P., Benamar, A., Detave, M., Moubarak, M., Hubert, V., Caus, T., Dupont, H., 2012. Rescue ECMO therapy in the treatment of refractory ARDS: 35 patients in a French hospital. Intensive Care Medicine 38, S259.
298. Zogheib, E., Guinot, P.G., Detave, M., Hubert, V., Badoux, L., Bernard, E., Urbina, B., Akinbusoye, O., Dupont, H., 2011. Passive leg raising can predict fluid responsiveness in patients placed on veno-venous extracorporeal membrane oxygenation (ECMO). Intensive Care Medicine 37, S92.
299. Zogheib, E., Piccardo, A., Guinot, P., Buchalet, C., Petiot, S., Moubarak, M., Hubert, V., Besserve, P., Benamar, A., Monconduit, J., Caus, T., Dupont, H., 2010. Extracorporeal oxygenation (ECMO) rescue in the treatment of severe ards with a refractory hypoxemia. Intensive Care Medicine 36, S358.
300. Zonies, D., Creutzenberg, M., Graf, B., -Stefan Hofmann, H., Bein, T., Fang, R., 2012. Oxygenation improvement with prone positioning in ECMO patients during post-traumatic ARDS. Critical Care Medicine 40, 51.
301. **GRADE Tables**
